# NeuroMatic: An Integrated Open-Source Software Toolkit for Acquisition, Analysis and Simulation of Electrophysiological Data

**DOI:** 10.3389/fninf.2018.00014

**Published:** 2018-04-04

**Authors:** Jason S. Rothman, R. Angus Silver

**Affiliations:** Department of Neuroscience, Physiology and Pharmacology, University College London, London, United Kingdom

**Keywords:** electrophysiology, patch clamping, data acquisition, data analysis, spike detection, spontaneous event detection, neural simulations, code:Igor Pro

## Abstract

Acquisition, analysis and simulation of electrophysiological properties of the nervous system require multiple software packages. This makes it difficult to conserve experimental metadata and track the analysis performed. It also complicates certain experimental approaches such as online analysis. To address this, we developed NeuroMatic, an open-source software toolkit that performs data acquisition (episodic, continuous and triggered recordings), data analysis (spike rasters, spontaneous event detection, curve fitting, stationarity) and simulations (stochastic synaptic transmission, synaptic short-term plasticity, integrate-and-fire and Hodgkin-Huxley-like single-compartment models). The merging of a wide range of tools into a single package facilitates a more integrated style of research, from the development of online analysis functions during data acquisition, to the simulation of synaptic conductance trains during dynamic-clamp experiments. Moreover, NeuroMatic has the advantage of working within Igor Pro, a platform-independent environment that includes an extensive library of built-in functions, a history window for reviewing the user's workflow and the ability to produce publication-quality graphics. Since its original release, NeuroMatic has been used in a wide range of scientific studies and its user base has grown considerably. NeuroMatic version 3.0 can be found at http://www.neuromatic.thinkrandom.com and https://github.com/SilverLabUCL/NeuroMatic.

## Introduction

Software packages for electrophysiological research are usually specialized to perform either data acquisition, data analysis or neuronal simulations, with little crossover in functionality. Hence, the majority of electrophysiologists find themselves acquiring data with one package, importing the acquired data into another package to perform data analysis, and using another package to perform simulations. They may use yet another package to create publication-quality graphics. A single unified package capable of performing acquisition, analysis and simulations, and creating publication-quality graphics, would substantially simplify this workflow, making it more efficient. Besides efficiency, however, such a unified package would enable a more integrated style of research. For example, online analysis during data acquisition can provide an immediate source of feedback for a stimulus protocol: recently acquired current-clamp data, such as action potentials, can be quickly repackaged as voltage-clamp commands to be applied to the same neuron to quantify the underlying currents. Similarly, trains of synaptic conductance waveforms can be simulated with different rise and decay kinetics and then sequentially injected into a neuron via dynamic clamp. A unified package would also facilitate the creation of comprehensive logs of the researcher's activity, from acquisition to analysis and simulation to the generation of tables and graphs. Such logs would improve research efficiency, documentation and reproducibility (Eglen et al., 2017; Munafò et al., 2017).

With this in mind, we developed NeuroMatic (RRID:SCR_004186), an integrated software package capable of performing a variety of patch-clamp recordings, data analysis routines and simulations of neural activity. The orchestration of such a wide range of software tools has been facilitated by an easy-to-use graphical user interface (GUI) with modular tab component that allows switching in and out the various tools while occupying minimal screen space. Moreover, NeuroMatic's back-end modular design facilitates expansion in almost all aspects, from adding hardware devices for acquisition to adding data analysis tabs and fit functions. At the heart of this back-end design is a hierarchical structure based on data folders, channels and sets that greatly simplifies the code structure.

NeuroMatic runs within Igor Pro (WaveMetrics, Portland, Oregon; https://www.wavemetrics.com; RRID:SCR_000325), here referred to as Igor, a platform-independent software environment for scientists and engineers that has an extensive library of built-in functions, a command line for executing built-in and user-defined functions, a history window for reviewing the user's command workflow and the ability to generate publication-quality graphics. It also has an easy-to-use programming/debugging environment with integrated help and documentation, which are especially useful for those needing to customize the functionality of NeuroMatic.

Since its original release, NeuroMatic has been used in numerous labs under a wide range of experimental paradigms (~4,500 downloads and 270 citations of the website to date). For acquisition, NeuroMatic has been used to perform glutamate uncaging (DiGregorio et al., 2007; Abrahamsson et al., 2012; Tran-Van-Minh et al., 2016), fluorescence recovery after photobleaching (FRAP; Rothman et al., 2016), optogenetic stimulation (Fukunaga et al., 2014; Pimentel et al., 2016), odor stimulation (Kohl et al., 2013), triggered communication with imaging software (Hofer et al., 2011; Fernández-Alfonso et al., 2014; Tran-Van-Minh et al., 2016), extracellular recordings of field potentials (Cao et al., 2013), continuous *in vitro* recordings of synaptic activity during triggered stimulation (Saviane and Silver, 2006b), continuous *in vivo* recordings during sensory stimulation (Arenz et al., 2008; Hofer et al., 2011; Rancz et al., 2011; Chabrol et al., 2015) and dynamic clamp (Rothman et al., 2009; Ward, 2012) via a dedicated analog signal processing board (Robinson and Kawai, 1993). For analysis, NeuroMatic has been used to perform spike detection (Kanichay and Silver, 2008; Rothman et al., 2009; Vervaeke et al., 2010; Ward, 2012; Chabrol et al., 2015), spontaneous event detection (Cooke and Woolley, 2005; Wulff et al., 2009; Kukley et al., 2010; Luikart et al., 2011; Schmeisser et al., 2012; Laprell et al., 2015), multiple-probability fluctuation analysis (MPFA; Silver, 2003; Chabrol et al., 2015), non-stationary noise (fluctuation) analysis (NSNA or NSFA; Traynelis et al., 1993; Hartveit and Veruki, 2007; Zonouzi et al., 2011; Coombs and Soto, 2016) and basic electrophysiological analysis such as current-voltage relations, paired-pulse ratios, rise times, calcium transient amplitudes, etc. (Nielsen et al., 2004; Saviane and Silver, 2006b; DiGregorio et al., 2007; Nolan et al., 2007; Soto et al., 2007; Branco et al., 2008; Nakamura et al., 2015; Tran-Van-Minh et al., 2016). More recently, NeuroMatic has been used to perform simulations of stochastic synaptic transmission (Rothman and Silver, 2014), short-term synaptic depression and facilitation (Schwartz et al., 2012; Rothman and Silver, 2014; Chabrol et al., 2015) and single-compartment integrate-and-fire (IAF) models (Schwartz et al., 2012; Chabrol et al., 2015). Several of these studies use NeuroMatic for nearly all aspects of acquisition and analysis.

Here, we detail the basic design of NeuroMatic version 3.0 and illustrate its use in acquisition, analysis and simulation. Source code and further documentation can be found at http://www.neuromatic.thinkrandom.com. Source code can also be found at a Github repository (https://github.com/SilverLabUCL/NeuroMatic) with instructions on how to contribute inside the README file.

## NeuroMatic overview

**Figure 1:**
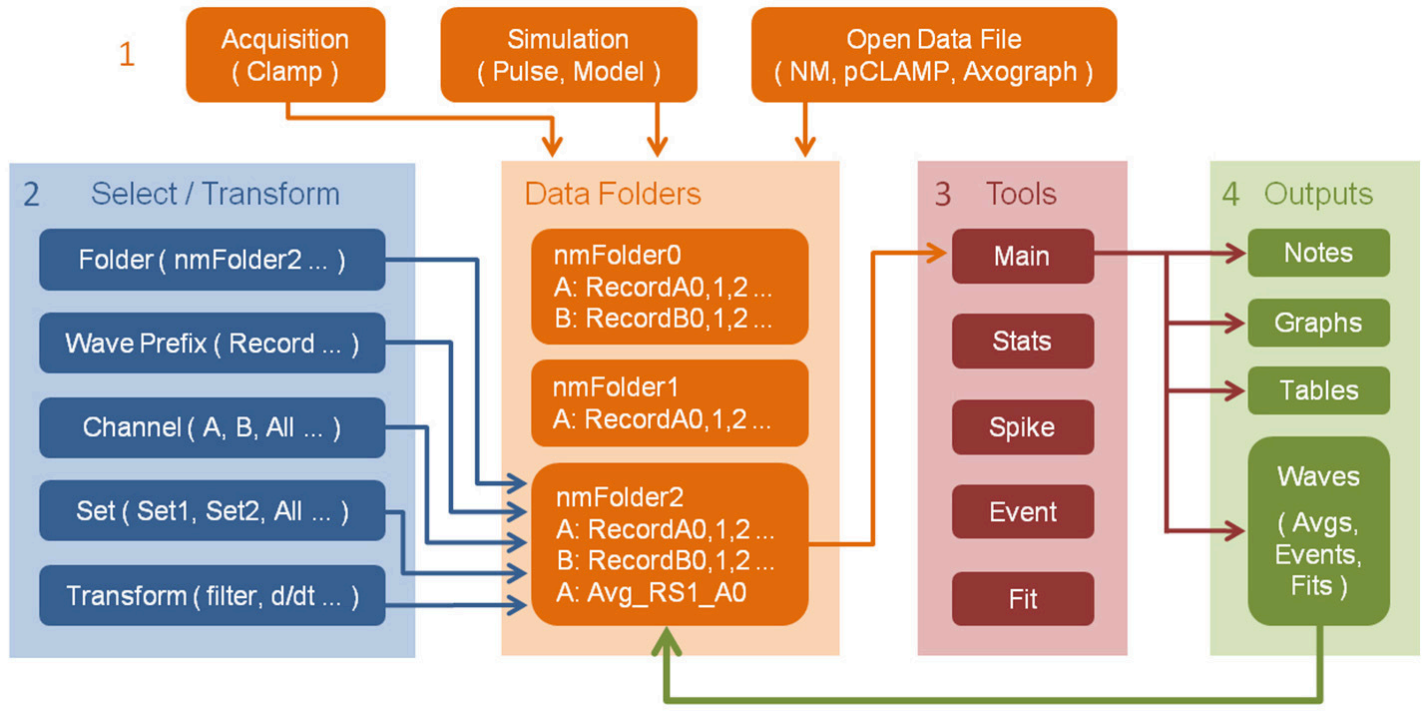
Workflow pattern within NeuroMatic enabling recursive analysis. Numbers denote workflow steps: **(1)** Work within NeuroMatic begins by either acquiring data via the Clamp tab, computing simulations via the Pulse or Model tab, or opening a pre-existing data file from a disk. The acquired/simulated/imported waves (i.e., time-series arrays) reside inside a NeuroMatic data folder (e.g., “nmFolder0”) and are named with the same wave prefix (e.g., “Record”), followed by a channel letter (e.g., A or B), followed by a sequence number (e.g., 0, 1, 2, etc.). **(2)** The user selects which data folder, waves, channels and sets to analyze (see Figure 2). The user can also select temporary transforms to apply to the waves, e.g., filter or baseline (see Figure 3). **(3)** The selected data is then analyzed using one or more of NeuroMatic's analysis tabs. **(4)** Outputs of the analysis often consist of waves that are displayed in graphs and tables. These output waves reside in the selected NeuroMatic data folder (green arrow) and can therefore be selected for further analysis via the *wave-prefix select* (2) (e.g., “Avg_”). Outputs also include notes printed to Igor's Command Window, including NeuroMatic function commands and analysis output, thereby providing documentation of the user's workflow and a means of creating user-defined functions. Notes are also attached to individual data waves if the waves are permanently transformed or created by an analysis function.

The typical workflow pattern within NeuroMatic is illustrated in Figure 1, numbered 1–4, which we summarize here. First, the user acquires data via the Clamp tab, or simulates data via the Pulse or Model tab, or imports one or more pre-existing data files from a disk. The acquired/simulated/imported data reside in Igor's local memory, inside one or more NeuroMatic data folders, as one-dimensional time-series arrays here referred to as “waves” to be consistent with Igor terminology. To allow analysis of multi-channel time-series waves, e.g., data simultaneously acquired from multiple analog channels of a data acquisition (DAQ) device, NeuroMatic automatically assigns the time-series waves to “channels,” denoted with letters A, B, C, etc. Second, using NeuroMatic controls, the user selects which data folder and waves to analyze and, if so desires, selects temporary transforms to apply to the waves (e.g., filter, baseline, differentiate). Third, the user analyzes the selected data via one or more of NeuroMatic's tab tools (Main, Stats, Spike, etc.). Fourth, the user visualizes/processes the results of the analysis, which are often output waves displayed in tables and graphs. Because these output waves are created inside the selected data folder (green arrow), they can be selected for further analysis; hence, NeuroMatic facilitates a recursive strategy of performing data acquisition, analysis and simulation. Moreover, because NeuroMatic function commands and analysis results are printed to Igor's Command Window (Menu/Windows/Command Window), a log of the user's activity is automatically generated, thus facilitating the documentation of complex workflow patterns and the generation of customized functions. A log of the user's activity is also maintained within the “note” memory of individual waves if, for example, the waves were permanently transformed (scaled, differentiated, etc.), or if the waves were created by an analysis function such as “Average” on the Main tab. A log of the user's activity is also maintained during data acquisition and automatically saved to disk for future reference.

**Figure 2:**
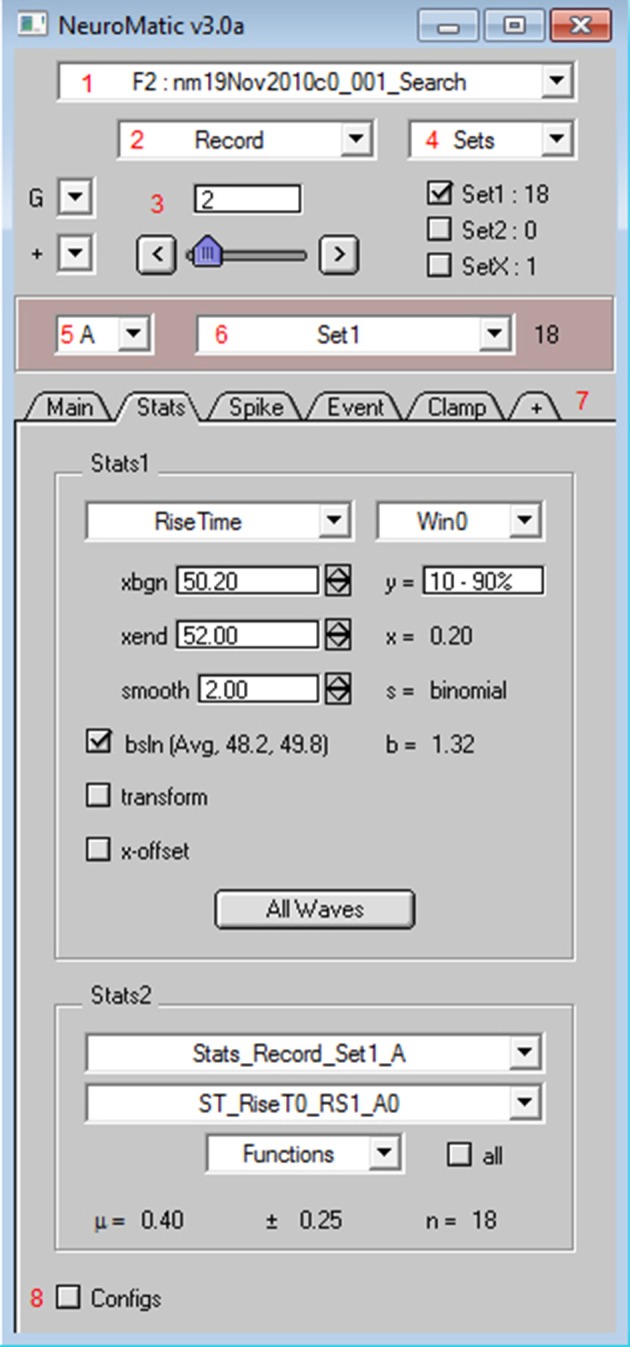
NeuroMatic's GUI. After installation, NeuroMatic's GUI automatically appears once Igor starts. Red numbers (added to this screen capture) denote GUI controls of interest: **(1)** The *folder select* is used to specify which NeuroMatic data folder to analyze, or to execute a folder function such as Open, Save or New. NeuroMatic data folders contain acquired/simulated/imported waves, plus all variables, waves and subfolders necessary to run NeuroMatic. **(2)** The *wave-prefix select* is used to specify which waves in the selected folder are to be processed. For example, “Record” selects waves named “RecordA0,” “RecordA1,” etc. Selected waves are sorted alphanumerically via their channel letter (e.g., “A”) and sequence number (0, 1, 2, etc.). **(3)** The *wave-number select* controls (set-variable, slider, < > buttons) are used to specify the wave sequence number to display in NeuroMatic's channel graphs (Figure 3). **(4)** Data waves can be categorized into sets (e.g., Set1 or Set2) via checkboxes or the sets Edit Panel. Total wave counts are displayed to the right of each set. Data waves can also be categorized into groups (e.g., Group0, Group1, etc.) via the groups (G) control. **(5)** The *channel select* is used to specify which data channel(s) to analyze (e.g., A, B, or All) for a multi-channel time series. **(6)** The *wave/set select* is used to specify which collection of waves to analyze (e.g., All or Set1 or Group0). The total wave count is displayed to the right. **(7)** NeuroMatic tabs can be switched in and out (+ tab) without the need to devote more screen space to new panels that clutter the desktop. Tabs perform analysis on the currently selected waves, or perform data acquisition (Clamp) or simulations (Pulse and Model), and are controlled by NeuroMatic's tab module. Here, the Stats tab is activated, showing results of a rise-time analysis of those waves in Set1. Users can also create their own tab using the Demo tab as a template (Supplementary Figure 1). **(8)** NeuroMatic configurations can be changed and saved for future use. Clicking the Configs checkbox activates NeuroMatic's configuration listbox for the currently selected tab. General NeuroMatic configurations are listed on the “NM” tab. More NeuroMatic functionality can be found under Igor's main menu (Menu/NeuroMatic), including keyboard shortcuts for repetitive tasks like changing the *wave-number select* (e.g., Ctrl+0 for next wave) or a wave's set categorization (e.g., Ctrl+1 for Set1 toggle). To install NeuroMatic simply place its package folder inside Igor's Procedures folder before starting Igor.

Nearly all of NeuroMatic's functionality just described is accessible via its main GUI, displayed in Figure 2, and can therefore be executed without the need to write code. However, all commands executed by the GUI appear in the command line/log enabling those unfamiliar with programming to learn the commands as they go along. The GUI is designed to be dynamic and extendable while occupying minimal screen space. At the top of the GUI are controls that allow users to execute various NeuroMatic functions pertaining to data folders and waves, and to select which waves are to be processed for visualization, manipulation, transformation and analysis. Here we discuss the NeuroMatic GUI controls labeled with red numbers in Figure 2, denoted below as (1), (2), etc.

At the top of NeuroMatic's GUI is the *folder select* control (1) which, when clicked, displays a menu list of all the NeuroMatic data folders that currently reside inside Igor's local memory. From this list the user selects which NeuroMatic data folder they wish to process. The list also contains several folder functions, such as “New,” “Save,” “Duplicate,” and “Open Data Files.” The latter option is used to open one or more NeuroMatic data folders saved on a disk (such as those created by the Clamp tab) or import another type of data file into a new NeuroMatic data folder. File formats supported for importing include Igor (binary or text), AxoGraph (https://axograph.com; RRID:SCR_014284), pCLAMP (https://www.moleculardevices.com; Axon Binary Format, ABF; RRID:SCR_011323), Excel, delimited text, HEKA Patchmaster (http://www.heka.com; RRID:SCR_000034), HDF5 (https://www.hdfgroup.org) and MATLAB (https://www.mathworks.com; RRID:SCR_001622), the last three being supported via Igor software extensions called XOPs. Hence, NeuroMatic can be used in conjunction with a wide range of acquisition and analysis software.

**Figure 3:**
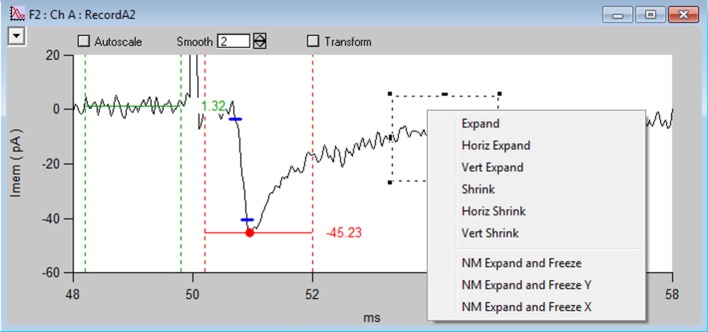
NeuroMatic's channel graph. Screen capture of the channel A graph (Ch A) for the Stats-tab rise-time analysis displayed in Figure 2, showing a voltage-clamp recording (RecordA2, black line) acquired from the cell body of a granule cell (GC) in a cerebellar slice, where extracellular stimulation of a single mossy-fiber (MF) synaptic input at 50 ms (see stimulus artifact, which has been clipped for display purposes) evoked an EPSC (inward current). Red circle and horizontal line denote the minimum of the EPSC computed within the dashed vertical red lines (*xbgn, xend*). An average baseline value (horizontal green line) has been computed before the EPSC within the dashed vertical green lines. *Blue dashes* denote where the EPSC reaches 10 and 90% of the peak amplitude from baseline. The time between these points is the 10–90% rise time (0.20 ms). Data was filtered by a binomial smooth function (2 smoothing operations). Note, each channel graph has options to apply temporary transforms to the data (top) such as filter, baseline, normalize and differentiate. Various display options are listed within the top-left popup control, such as overlay, grid lines and axis scaling. More options are listed by clicking inside an Igor marquee (dashed square), created by clicking and dragging the mouse diagonally to frame a region of interest. Data was acquired via the Clamp tab from a cerebellar slice from a P24 mouse, where access resistance (R_a_) = 14 MΩ, membrane capacitance (C_mem_) = 2.5 pF and the holding potential (V_hold_) = −76.3 mV.

Within each NeuroMatic data folder are the waves of interest. Those waves constituting a time series must be named with the same prefix in order for NeuroMatic to recognize the series. Data waves acquired via NeuroMatic's Clamp tab, for example, are by default named “RecordA0,” “RecordA1,” “RecordA2,” etc., where “Record” is the wave prefix, “A” denotes the acquisition channel (A, B, C, etc.), followed by a repetition series number (0, 1, 2, etc.). To analyze a time series, the user specifies which waves are to be processed via NeuroMatic's *wave-prefix select* (2), e.g., “Record.” Once specified, NeuroMatic determines the number of channels in the time series, applies the wave-name convention described above and displays the waves in one or more channel graphs (Figure 3). The user specifies the series number to be displayed via the *wave-number select* controls (3), e.g., “2.”

One of the more practical aspects of NeuroMatic is the ability to categorize waves into sets via checkboxes and functions (4). Default sets are Set1, Set2 and SetX, where SetX is a special set used to exclude data from analysis, such as recordings corrupted by spurious noise. Waves can also be categorized into groups (G), which are disjoint sets that can be used to analyze data acquired in a repeating sequence, such as a current-voltage relation repeated multiple times. Once sets and/or groups are defined, the *channel select* (5) is used to designate which channel to analyze, e.g., “A,” and the *wave/set select* (6) is used to designate which set of waves or combination of sets to analyze, including the default “All” option for all waves.

Within individual channel graphs (Figure 3) users also have the option of applying transformations to their data, such as filter, baseline, normalize differentiate, running average, etc. These transformations can be turned on and off.

NeuroMatic's acquisition/analysis/simulation tools are activated via a tab bar (7). In Figure 2, the Stats tab has been activated and a Stats window (Win0) has been configured to compute the 10–90% rise time of an excitatory postsynaptic current (EPSC). Results of the analysis for the selected wave number are displayed on the right of the Stats tab (x = 0.20 ms) as well is in the channel graph (Figure 3). If one then wants to compute the same analysis on all selected waves, i.e., those waves captured by the folder (1), wave-prefix (2), channel (5), and wave/set (6) selects, one simply needs to click the “All Waves” button. At this point, NeuroMatic steps through the list of selected waves and computes the same Stats analysis, saving results to new waves, referred to as Stats waves, created within the selected folder. These Stats waves can be displayed in graphs and tables and used for further analysis such as computing means, variances, histograms, via the Stats2 tab controls. The Stats waves can also be used to create sets via the Stats2 Inequality function; for example, if a user wishes to categorize all waves with a rise time <0.6 ms as Set1. Other tabs include the Clamp tab which performs data acquisition, the Main tab which performs basic wave manipulations (graph, edit, copy), transformations (redimension, baseline, scale) and analysis (average, sum, histogram), the Spike tab which performs spike threshold detection and analysis (raster plots, peristimulus time histograms, inter-spike-interval histograms), the Event tab which performs event detection using a sliding-window threshold-detection algorithm (Kudoh and Taguchi, 2002) or a match-template algorithm (Clements and Bekkers, 1997), the Fit tab which performs curve fitting, the Pulse tab which performs waveform generation/simulation, including synaptic waveforms such as EPSCs, the Model tab which performs single-compartment IAF and Hodgkin-Huxley-like simulations, and the Demo tab (Supplementary Figure 1) which can be copied and modified to create customized user tabs.

Configurations that control NeuroMatic's default behavior can be edited by clicking the Configs checkbox (8). The configurations are displayed and edited in a ListBox control and can be saved for future use (Menu/NeuroMatic/Configurations/Save/All). If the configurations are saved inside NeuroMatic's package folder they will automatically be loaded once NeuroMatic starts.

## NeuroMatic design

### Data folders

**Figure 4:**
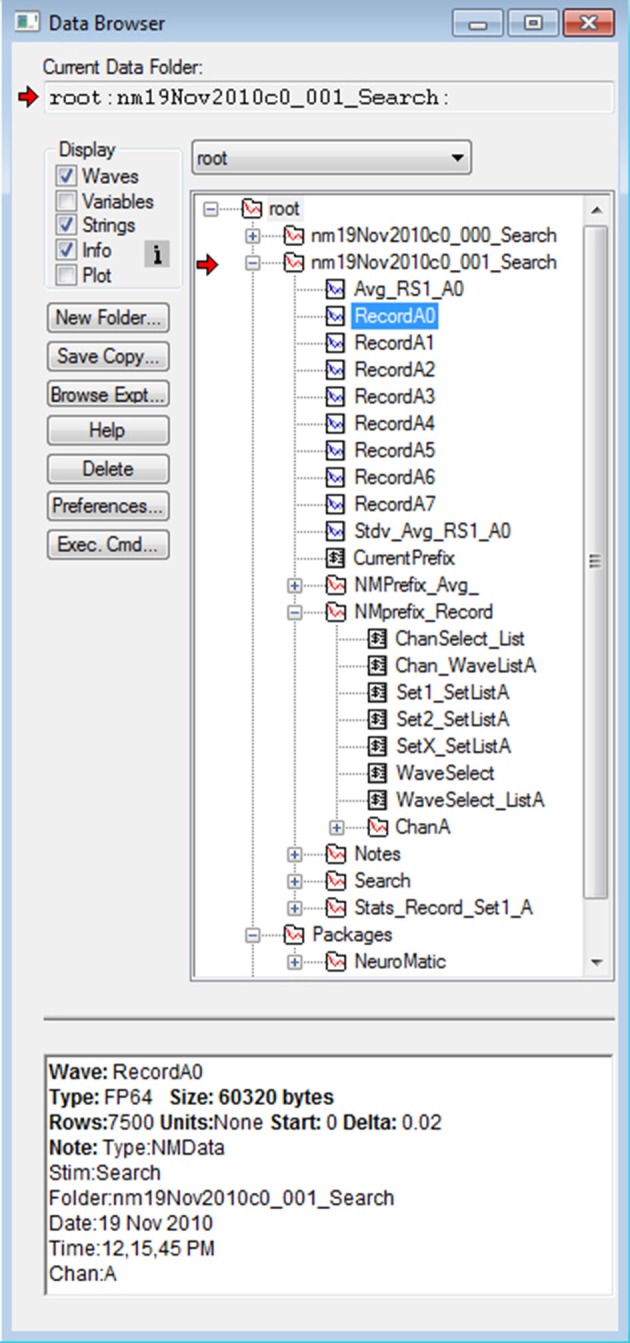
NeuroMatic's hierarchical folder structure. Screen capture of Igor's Data Browser (Menu/Data/Data Browser), a useful tool for navigating through NeuroMatic's data folder hierarchy. Here, all folders and variables were created by NeuroMatic inside Igor's local-memory root directory. The top two folders beginning with “nm” were created by the Clamp tab after executing a stimulus protocol called “Search.” *Red arrow* denotes the folder selected for processing (Figure 2, control 1) which was clicked open for browsing. Inside the folder are waves with prefix “Record” and channel letter “A,” and a string variable *CurrentPrefix* containing the value of the *wave-prefix select* (Figure 2, control 2) whose current value is “Record,” in which case the prefix subfolder “NMPrefix_Record” is activated. This prefix subfolder has been opened, showing string variables containing wave name lists for channel A, values for the *channel* and *wave/set selects* (Figure 2, controls 5 and 6), wave name lists for Set1, Set2 and SetX, and a subfolder (ChanA) containing channel graph information like axis scales and wave transformations. Other subfolders were created by the Clamp tab (Notes and Search), Stats tab (Stats_Record_Set1_A) and by entering “Avg_” for the *wave-prefix select* (NMPrefix_Avg_). Here, wave RecordA0 has been selected (blue), revealing its “Info” at the bottom, including Igor information and NeuroMatic acquisition and analysis notes; this information can be printed to the Command Window or an Igor notebook via the Main tab (Display/Print Notes; Supplementary Figure 4). Note, some waves, string variables and wave notes have been removed for display purposes, and numeric variables are not shown. NeuroMatic's private data for nearly all its functionality is located in the root:Packages:NeuroMatic folder.

The basic structural element of NeuroMatic is the data folder, which resides in Igor's local memory known as the “root” directory (Figure 4). NeuroMatic data folders contain the acquired/simulated/imported waves of interest (e.g., RecordA0, RecordA1… or Sim_Vmem_A0, Sim_Vmem_A1…) and analysis output waves created by NeuroMatic (e.g., Avg_RS1_A0 and Stdv_Avg_RS1_A0). They also contain a string variable called *CurrentPrefix* which stores the current value of the *wave-prefix select* (e.g., “Record”). All control variables related to *CurrentPrefix* are then stored inside a *prefix subfolder* whose name begins with “NMPrefix_” and ends with the value of *CurrentPrefix* (e.g., “NMPrefix_Record”). These control variables include values for the *wave-number, channel* and *wave/set selects*, and the wave names listed within the sets. The *prefix subfolder* also contains one or more *channel subfolders* (e.g., ChanA) that store channel graph variables related to the value of *CurrentPrefix*. If the data folder was created via the Clamp tab, it will also contain a *stimulus subfolder*, which is a copy of the stimulus protocol used to acquire the data, and a *notes subfolder* containing user notes saved during acquisition. The data folder may also contain subfolders created by one or more NeuroMatic tabs (e.g., Stats_Record_Set1_A or Fit_Line_Record_All_A) that contain results of analysis.

Using functions listed within NeuroMatic's *folder select* control (Figure 2, control 1), NeuroMatic data folders can be renamed, copied and merged. The folders can be readily shared between different Igor experiments by saving them to disk and opening in another Igor experiment, or dragging them from one Igor experiment to another via Igor's Data Browser (Figure 4; “Browse Expt…”).

### Modules

Most of NeuroMatic's functionality is coordinated by a collection of software modules that perform logically discrete functions (Supplementary Figure 2A), including modules for tabs, folders, channel graphs, analysis graphs and tables, history notes, configurations, data importing, sets, fit functions, pulse waveforms and Hodgkin-Huxley-like models. Moreover, each tab is a module in itself, enabling/disabling tab controls, creating waves, global and configuration variables, and updating the analysis and display of the currently selected channel wave (Figure 3). The Demo tab, for example, is the simplest tab and is designed to form the framework of user-defined tabs (Supplementary Figure 1). The tab's code (Menu/NeuroMatic/Procedures/Tabs/NM_DemoTab.ipf) can be copied, pasted into a new Igor procedure file (Menu/Windows/New/Procedure…) and modified by just two search-and-replace commands to create a new NeuroMatic tab (see NM_DemoTab.ipf for more details). The tab's code contains functions for creating button and setvariable controls, prompts for user input, and performing analysis on the currently selected folder, wave prefix, channel and sets (e.g., NMDemoLoop). All of these functions can be tailored to suit the user's needs. The Clamp tab, on the other hand, is the most complicated tab, containing an assemblage of modules for communicating with DAQ devices (NI-DAQ and ITC), reading telegraph amplifier inputs (Axopatch, MultiClamp, Dagan and Alembic), computing online analysis, creating notes, log files, stimulus protocols and configurations (Supplementary Figure 2B). Despite its more complicated structure, however, a few aspects of the Clamp tab can be extended to include new functionality. Source code for computing online analysis, for example, can be used to create customized online analysis routines that run before, during or after data acquisition (Menu/NeuroMatic/Procedures/Clamp/NM_ClampUtility.ipf), and source code for NeuroMatic's “demo” acquisition mode can be used as the framework for acquiring data from other DAQ devices (Menu/NeuroMatic/Procedures/Clamp/NM_ClampAcquireDemo.ipf).

The source code for each NeuroMatic module is usually contained within a single Igor procedure file that resides inside NeuroMatic's package folder. These procedure files can be easily accessed via NeuroMatic's menu (Menu/NeuroMatic/Procedures) and extended to provide custom functionality. NeuroMatic's file import module, for example, can be extended to support different file formats, its Model module can be extended to included different Hodgkin-Huxley-like models, its Pulse and Fit modules can be extended to include user-defined functions, and, as stated above, its Clamp module can be extended to include online analysis via user-defined functions, acquisition from different DAQ devices and telegraphing from different patch-clamp amplifiers.

All of NeuroMatic's private data that control its GUI, menu, prompts, windows, etc., are stored within Igor's local root directory in a folder called Packages (Figure 4; root:Packages:NeuroMatic), as recommended by WaveMetrics. Data within the NeuroMatic package folder is further subdivided via subfolders with respect to tabs and configurations. Functions for managing NeuroMatic's package folder can be found in NeuroMatic's main procedure file (Menu/NeuroMatic/Procedures/Misc/NM_Main.ipf).

### Macro loops

As evident in Figure 4, NeuroMatic organizes its data via the following hierarchical structure from top to bottom: folders, wave prefixes, channels and sets. Likewise, many of NeuroMatic's back-end data analysis functions contain the same hierarchical structure. Most of the functions on the Main tab, for example, use nested for-loops that follow this structure (Supplementary Figure 3). The nested for-loops reside in a core NeuroMatic function called NMLoop which receives a string-variable list for each of its input parameters (e.g., “nmFolder0,” “Record,” “A;B;C;” and “Set1;Set2;” for folders, wave prefixes, channels and sets, respectively). Each for-loop iterates over the items of its input list, having one or more items. At the center of the nested loops, individual items for a folder, wave prefix, channel and set are passed to a designated wave processing function. The ability to pass one or more items as an input parameter to NMLoop greatly increases its computational power and reduces the amount of source code needed to create user-defined functions, especially if one needs to compute the same analysis on multiple folders, wave prefixes, channels or sets.

The design of the core NMLoop function (Supplementary Figure 3) makes the extension of NeuroMatic's wave processing capacity fairly straightforward. One only needs to create a wrapper function that passes the various parameter lists to NMLoop (e.g., MyFxn) and a wave processing function that executes a desired process on the collection/list of waves defined by a given folder, wave prefix, channel and set (e.g., MyFxn2). Demo functions containing the essential elements of the wrapper and wave processing functions (NMDemoLoop and NMDemoLoop2) can be found in the NeuroMatic procedure file NM_DemoTab.ipf (Menu/NeuroMatic/Procedures/Tabs). These functions can be copied, renamed and edited to extend NeuroMatic's wave processing capacity.

### Command history, wave notes and acquisition metadata

A core feature of Igor is its Command Window (Menu/Windows/Command Window) where executed Igor commands are automatically printed. Results of analysis like curve fitting and wave statistics (e.g., Igor command WaveStats) are also printed to this window. Hence, the Command Window provides a valuable source of documentation of the user's workflow.

**Figure 5:**
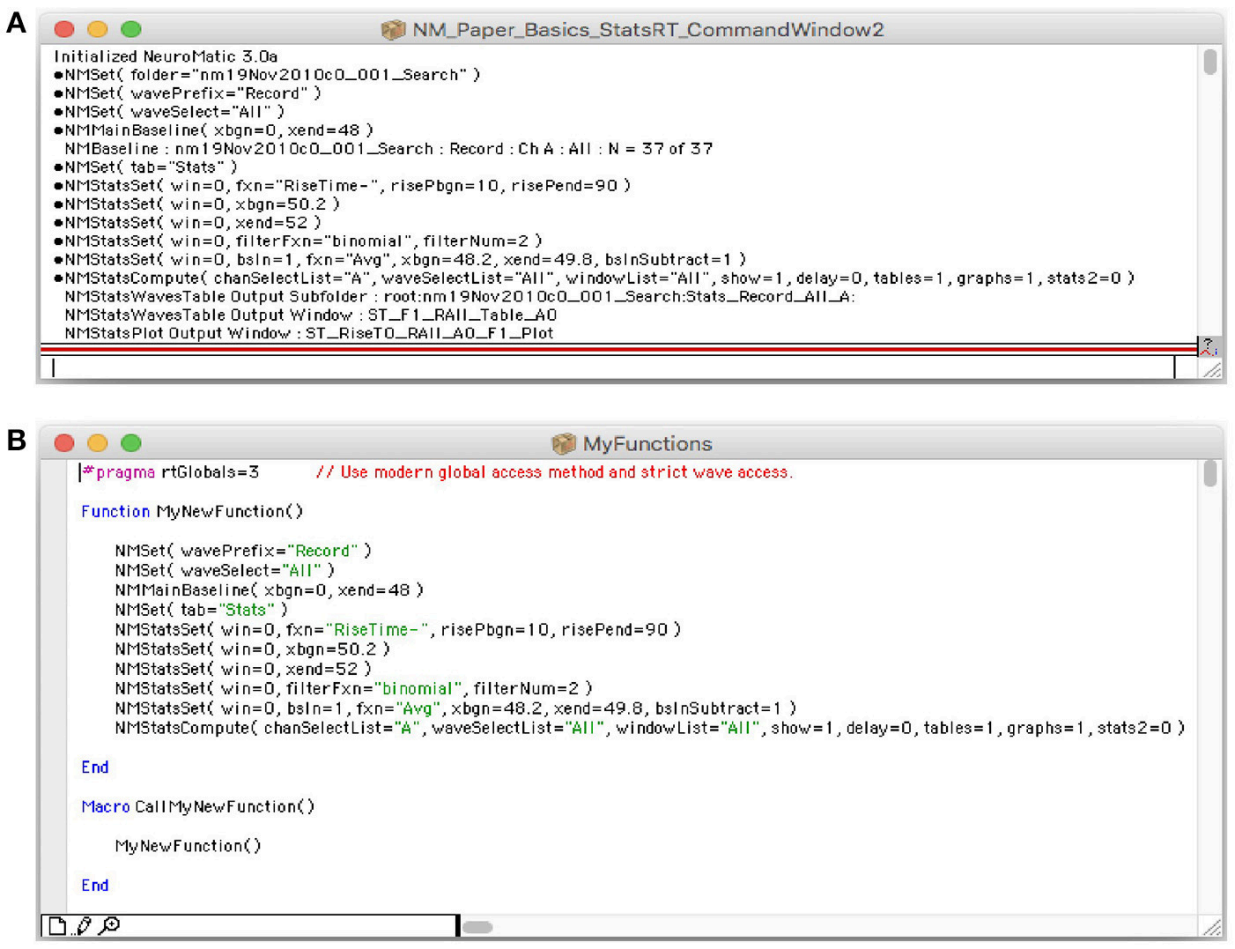
NeuroMatic commands and notes printed to Igor's Command Window. **(A)** Screen capture of Igor's Command Window (Menu/Windows/Command Window), a useful tool that creates a historical log of the researcher's workflow, from acquisition to analysis and simulation. Both Igor and NeuroMatic print function commands and analysis/transform notes to this window. Function commands are preceded by a small circular bullet. Analysis/transform notes sometimes appear after the function command. NeuroMatic commands can also be printed to an Igor notebook (GUI/Configs/NM/CmdHistory/Notebook). **(B)** Screen capture of an Igor procedure window (Menu/Windows/New/Procedure…) named “MyFunctions” containing a function called “MyNewFunction.” NeuroMatic function commands were copied from Igor's Command Window **(A)** and pasted into this function. A simple macro named “CallMyNewFunction” was created to call MyNewFunction. Macros are automatically listed in Igor's Macros menu where they can be readily selected and executed. In this way users can create functions of repetitive tasks that need to be performed on multiple data files. Creating such user-defined functions improves research efficiency, documentation and reproducibility.

Similar to Igor, NeuroMatic prints many of its function commands and analysis results to the Command Window (Figure 5A). NeuroMatic function commands usually contain the letters “NM.” Because the function commands are in an executable format (i.e., they contain a list of the input parameters within parentheses), users can copy these commands and enter them into the Igor command line (immediately below the history window) or into their own Igor functions (Figure 5B; also see the Igor procedures provided in the Supplementary Material) to replicate what was executed via NeuroMatic's GUI. Hence, the creation of customized functions using NeuroMatic commands can be as easy as copying and pasting. Following the function commands, NeuroMatic will print the results of analysis or transforms to the Command Window (Figure 5A), including information about the data folder, wave prefix, channel and waves/sets that have been processed, or the names of subfolders, graphs and tables created by NeuroMatic.

Besides Igor's Command Window, data waves created and processed by NeuroMatic contain history information (i.e., wave notes) pertaining to acquisition, analysis and simulation. These wave notes can be viewed in Igor's Data Browser (Figure 4, “Info”) or printed to the Command Window or an Igor notebook (GUI/Main/Display/Print Notes; Supplementary Figure 4). The combination of Igor's Command Window and wave notes thus provides a valuable log of the user's activity.

As mentioned above, if a NeuroMatic data folder was created via the Clamp tab, it will contain a copy of the stimulus folder used to acquire the data. Information stored inside the stimulus folder, such as input/output configurations (ADC/DAC/TTL), timing (acquisition mode, interludes, repetitions) and pulse waveforms can be quickly retrieved via NeuroMatic menu options (Menu/NeuroMatic/Clamp Data) or viewed within Igor's Data Browser (Figure 4). The NeuroMatic data folder will also contain the user's notes saved during acquisition (inside the Notes subfolder) and can be quickly retrieved as well (Menu/NeuroMatic/Clamp Data/Notes Table). These notes can also be viewed within the Clamp log file, which contains a log of the user's acquisition history, including protocol names and time stamps. The log file is saved to a user-specified disk in the same location as the data files and is opened like any other data file (GUI/Folder Select/Open Data Files).

## NeuroMatic use examples

Here, we present examples of acquisition, analysis and simulation of electrophysiological data using NeuroMatic. The examples are intended to illustrate the wide range of NeuroMatic's functionality and the recursive workflow pattern illustrated in Figure 1. To demonstrate how relatively simple it is to generate user-defined functions using NeuroMatic and Igor function commands, we provide our own user-defined functions for performing the data analysis and simulations and generating the figures for each example. These functions are saved within Igor procedure files which can be loaded and compiled within Igor, and are provided as Supplementary Material. All of the data used in these examples comes from whole-cell recordings from cerebellar granule cells (GCs) in slice tissue at physiological temperatures. More how-to-use-NeuroMatic examples can be found elsewhere (Hartveit and Veruki, 2007; Coombs and Soto, 2016).

### Acquisition of whole-cell current-clamp data with online spike analysis

**Figure 6:**
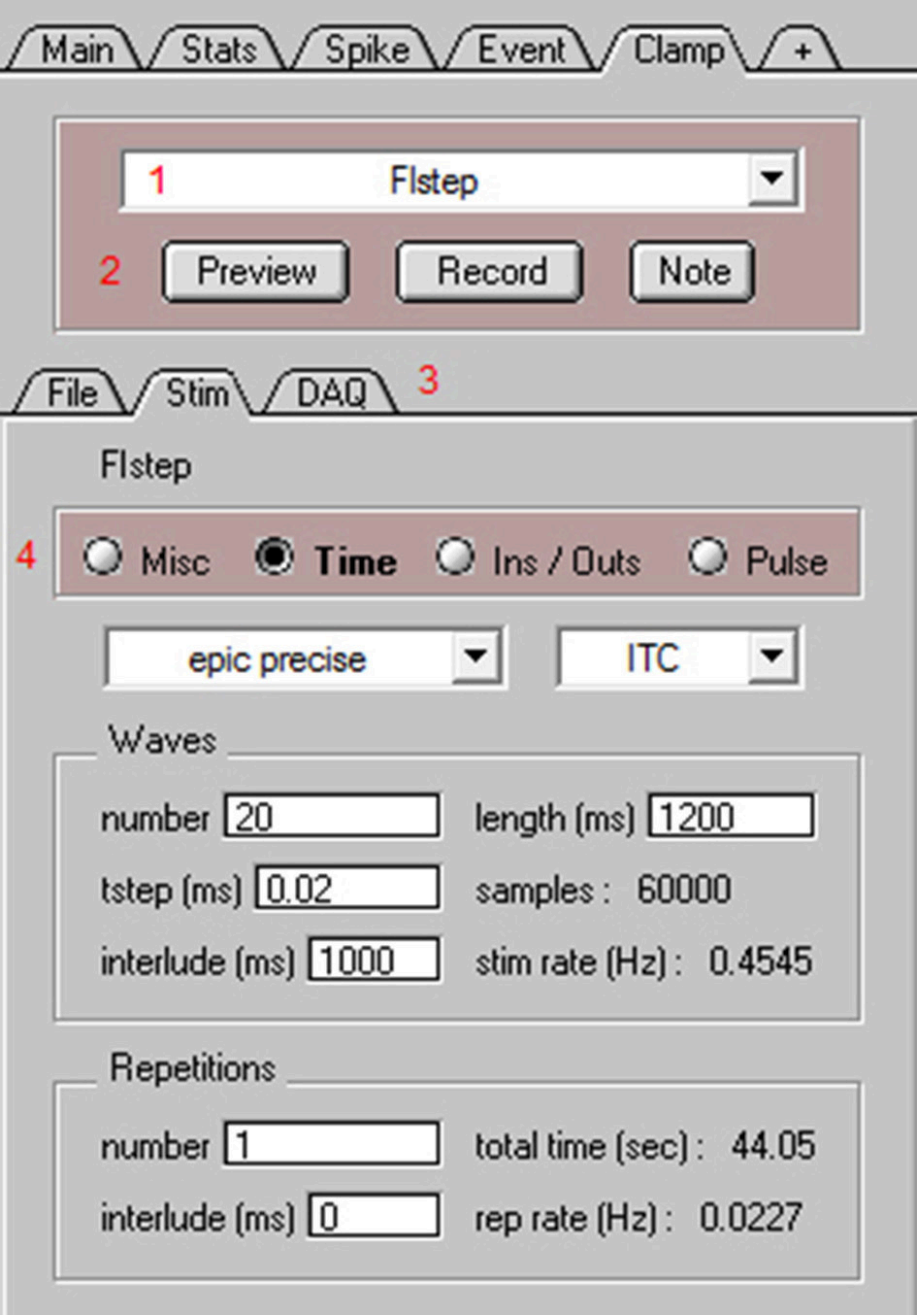
NeuroMatic's Clamp tab for data acquisition. Screen capture of the bottom of NeuroMatic's main control panel after activating the Clamp tab (Figure 2, control 7). The Clamp tab is used to create input/output configurations for either NI-DAQ or ITC devices, and to create and execute stimulus protocols for data acquisition. Red numbers denote controls of interest: **(1)** the *stimulus select* for specifying which stimulus protocol to activate (e.g., FIstep), or to perform a stimulus function such as Open, Save, New, Copy; **(2)** the *Preview* and *Record* buttons for executing the currently selected stimulus protocol, and the *Note* button for saving time-stamped notes to the data and log files; **(3)** Three Clamp sub-tabs including the *File* tab for setting parameters pertaining to the data folder/file name format (e.g., date prefix, cell and sequence number, external directory path), notes (e.g., name, lab, experiment title) and log files, the *Stim* tab (see below) and the *DAQ* tab for setting parameters pertaining to the DAQ device (e.g., ADC channel number, name, units, scale factor; see Supplementary Figure 5); **(4)** the *Stim* tab for configuring the currently selected stimulus protocol via four radio buttons: *Misc* for configuring stimulus chains, online Stats and Spike analysis, macros for reading temperature, telegraphed parameters, randomizing sequence order, computing running averages or electrode resistance; *Time* for specifying the acquisition mode (episodic, epic precise, continuous, triggered), sample time (tstep), number of waves, repetitions and interludes; *Ins*/*Outs* for selecting which ADC, DAC and/or TTL configurations to use; and *Pulse* for generation of voltage, current, conductance and TTL output waveform commands.

Before using NeuroMatic's Clamp tab (also known as Nclamp) for data acquisition, three initial tasks must be performed: (1) An XOP (software extension) that allows Igor to communicate with the given DAQ device (NI-DAQ or ITC) must be placed inside Igor's Extensions folder. The XOP for NI-DAQ devices is provided by WaveMetrics and the XOP for ITC devices is provided by HEKA Instruments. (2) Configurations pertaining to the DAQ device must be entered via the Clamp/DAQ tab (Figure 6; Supplementary Figure 5). Configurations for ADC inputs and DAC/TTL outputs include the device channel number, scale conversion factor, units (e.g., “pA” or “mV”) and a unique ID name (e.g., “Icmd” or “Vmem”). Automatic scaling of the ADC inputs can also be configured via telegraph gain inputs from the following amplifiers: Axopatch 200A and 200B, MultiClamp 700A and 700B, Axopatch 1D, Dagan 3900A, Alembic VE2. MultiClamp telegraphing requires installation of the AxonTelegraph XOP provided by WaveMetrics (search “Installing The AxonTelegraph XOP” in the Igor Help Browser; Menu/Help/Igor Help Browser). (3) An external directory path identifying the location on a disk where NeuroMatic data folders are to be saved must be entered on the Clamp/File tab (“save to”). Additional optional tasks include: (1) Editing the file header Notes on the Clamp/File tab, such as the user's name, lab's name, and experiment title. This is highly recommended as NeuroMatic saves this information inside each data folder it creates as well as the Clamp log file. (2) Choosing the file format for saving NeuroMatic data folders on the Clamp/File tab (e.g., Igor binary or HDF5). (3) Saving all configurations for future use (Menu/NeuroMatic/Configurations/Save/All). If configurations are saved inside NeuroMatic's procedure folder (default option) the configurations will automatically be loaded once NeuroMatic starts.

The next step to performing data acquisition is to create one or more stimulus protocols. Using the Clamp stimulus protocol control (Figure 6, control 1) the default stimulus protocol “nmStim0” can be renamed, or a new protocol created. The Clamp/Stim radio buttons (Figure 6, control 4) can then be used to choose the desired ADC input and DAC/TTL output configurations (Ins/Outs), set the acquisition timing parameters (Time; wave length, sample rate, time interlude, repetitions), create DAC and TTL output commands (Pulse), and configure online stats and spike analysis and any runtime analysis such as reading the temperature from an ADC input (Misc).

**Figure 7:**
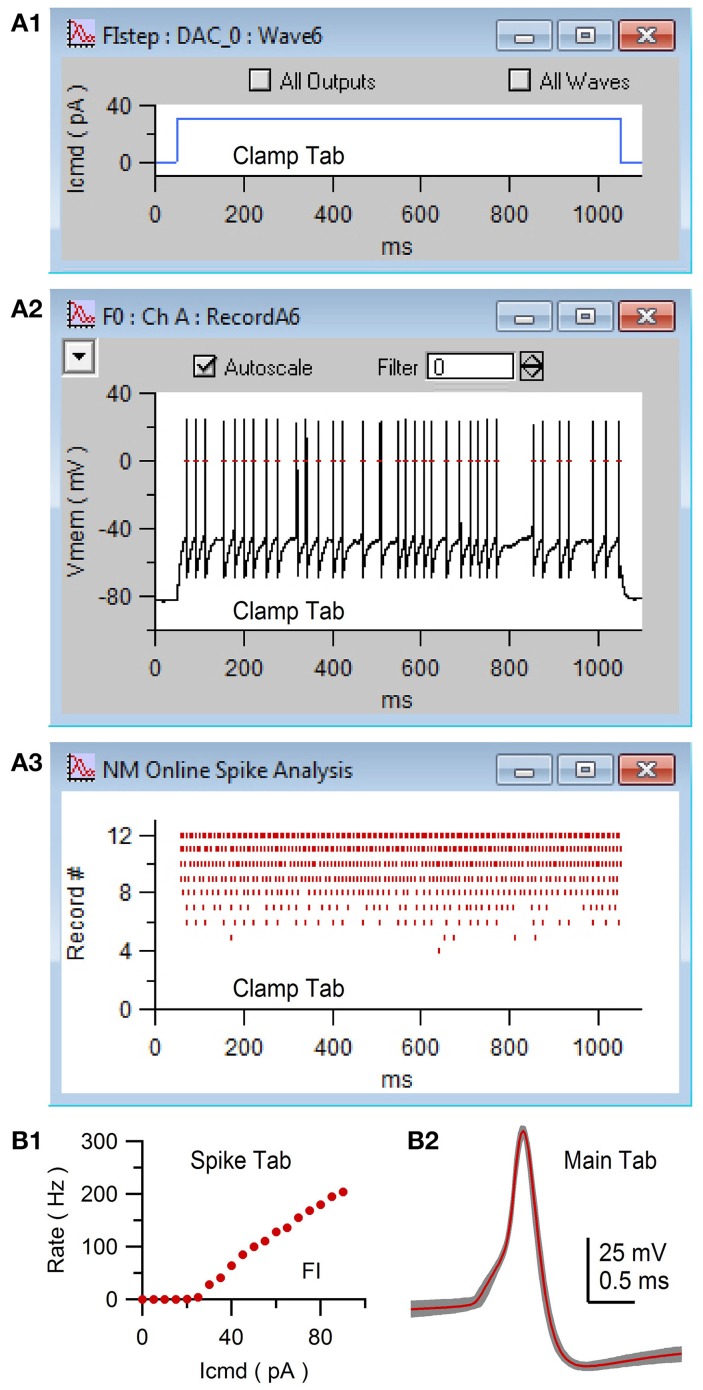
Acquisition of whole-cell current-clamp data with online spike detection via NeuroMatic's Clamp tab. Clamp tab acquisition of the membrane potential (V_mem_) of a GC. The stimulus protocol was called FIstep (see Figure 6) consisting of 20 consecutive step current commands (I_cmd_) with increasing amplitude (+5 pA). **(A1)** Plot of the seventh I_cmd_ (DAC wave #6) consisting of a 1 s step to 30 pA. The plot was created via the Clamp/Stim/Pulse/Plot button. **(A2)** Channel graph display of V_mem_ in response to I_cmd_ in **(A1)**. Red dashes denote action potentials detected by NeuroMatic's online spike detector (threshold = 0 mV), activated via the “spike” checkbox on the Clamp/Stim/Misc tab. **(A3)** Spike raster plot of NeuroMatic's online spike detector for recordings #0–12. **(B1)** Spike-tab analysis after acquisition. Spike rate versus I_cmd_ (FI relation) computed via the “Avg Rate” button. **(B2)** Detected spikes were copied to new waves with prefix “Spike0” via the “Spikes 2 Waves” button on the Spike tab. Shown here are the copied spikes overlaid (gray; *n* = 268) and their average (red) computed via the Main tab. Experimental details: cerebellar slice from a P35 rat, R_a_ = 17.5 MΩ, C_mem_ = 2.4 pF.

For the use example here, we created a current-clamp stimulus protocol called “FIstep” (Figure 6). On the Clamp/Stim/Ins/Outs tab, the “Vmem” ADC input and “Icmd” DAC output were selected. This input and output were previously configured and saved via the Clamp/DAQ tab to record from an ITC device's ADC channel 0, which was connected to the output of an Axopatch 200B amplifier (Molecular Devices), and to send a current command waveform to the ITC device's DAC channel 0, which was connected to the external command input of the same amplifier. On the Clamp/Stim/Time tab, the protocol was configured for acquisition from an ITC board using the episodic precise mode: episodic in that there is a 1 s interlude between acquisition of data waves of 1.2 s length (60,000 samples of 20 μs) and precise in that the interlude is timed by the ITC device rather than Igor's microsecond timer. In order to create consecutive current commands with increasing amplitude, the number of waves was set to 20. Using the Clamp/Stim/Pulse tab, a square step of 1 s duration was added (“+”) to all 20 current command outputs (Icmd). A delta of 5 was entered for the amplitude so that consecutive commands increase in size by 5 pA. The resulting pulse configuration had the following notation: “wave = all;pulse = square;amp = 0,delta = 5;onset = 50;width = 1,000;”. Next, online spike detection was turned on by clicking the “spike” checkbox on the Clamp/Stim/Misc tab and a threshold of 0 mV was entered. Finally, whole-cell access to a GC was obtained in slice tissue. The mode of the Axopatch 200B amplifier was set to I-Clamp and the FIstep protocol was executed by clicking “Record” (Figure 6, control 2). During acquisition, NeuroMatic displayed the cell's membrane response to current injection in a channel graph and denoted spike threshold crossings with red dashes (Figure 7A2). Spike times were simultaneously displayed in a spike raster plot (Figure 7A3). After acquisition, NeuroMatic's Spike tab was used to compute a relation between the spike output rate and the amount of current injection (Figure 7B1; FI relation) by clicking “Avg Rate.” Detected spikes were then copied to new waves with prefix name “Spike0” by clicking “Spikes 2 Waves,” and the average of the resulting spike waves was computed via the Main tab (Figure 7B2).

### Acquisition, detection and analysis of spontaneous EPSCs

To acquire a long stretch of continuous membrane current containing spontaneous EPSCs, a protocol called “Minis” was created using the Clamp stimulus protocol control (Figure 6, control 1, “New”). On the Clamp/Stim/Ins/Outs tab, the “Imem” ADC input was selected, which was configured to record from a NI-DAQ device's ADC channel 0, which was connected to the output of an Axopatch 200B amplifier. On the Clamp/Stim/Time tab, “continuous” mode was selected and the wave length was set to 1 s (50,000 samples of 20 μs). Repetitions was set to 300 with 0 s interlude, giving a maximum acquisition time of 300 s. Finally, using an Axopatch 200B amplifier, whole-cell access to a GC was obtained in slice tissue in voltage clamp (V-clamp) mode, with the holding potential (V_hold_) set to −80 mV. The Minis protocol was activated by clicking “Record” on the Clamp tab. After 170 s, acquisition was stopped by clicking “cancel” on NeuroMatic's progress window. Using the Main tab, the resulting 170 waves were concatenated into a single wave with prefix “C_Record” (GUI/Main/Edit/Concatenate). This prefix was selected using NeuroMatic's *wave-prefix select* (Figure 2, control 2).

**Figure 8:**
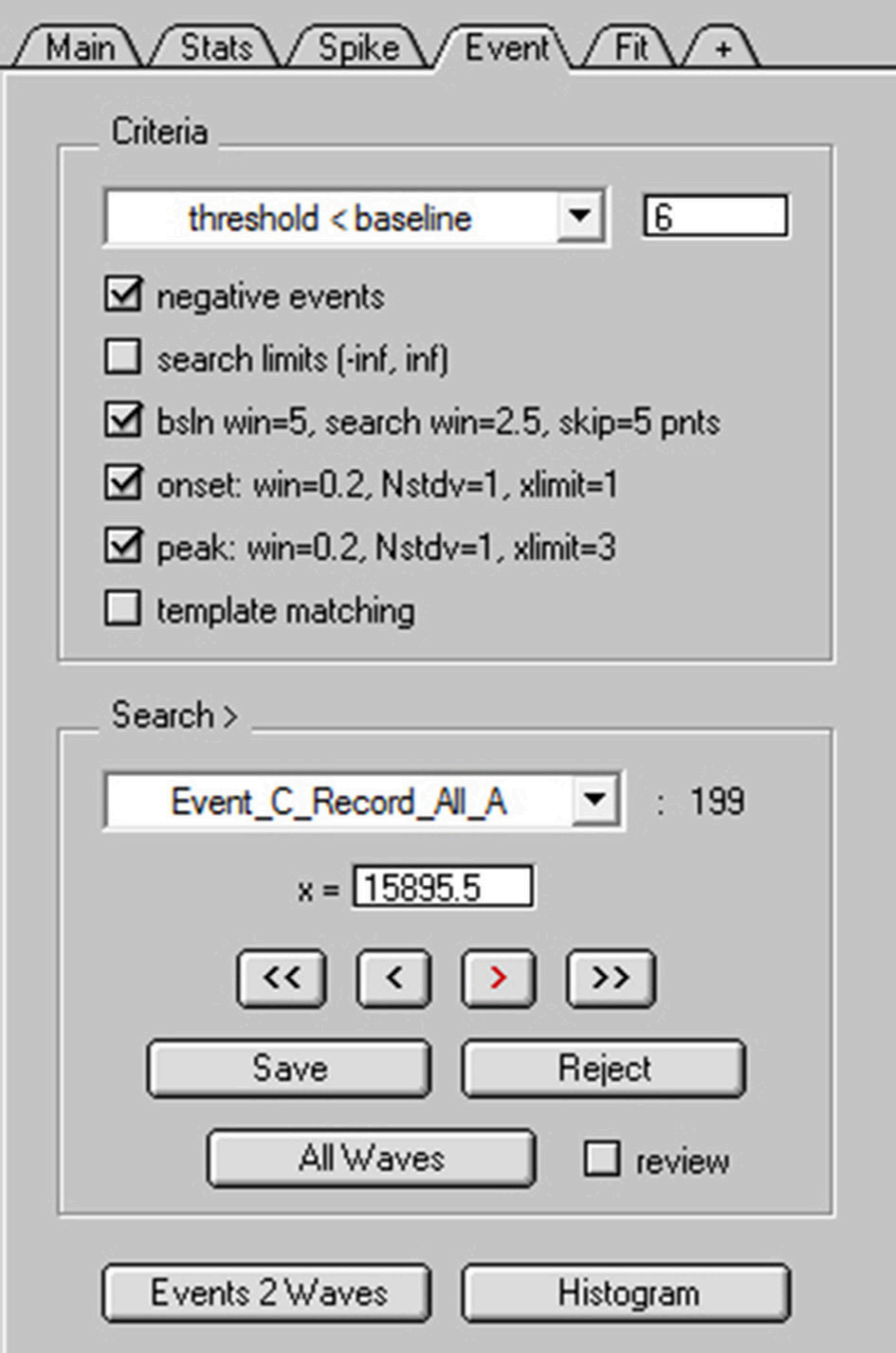
NeuroMatic's spontaneous event detection. Screen capture of the criteria and search controls for the Event tab during spontaneous EPSC detection. Criteria controls **(Top)** define which search algorithm to use (threshold-level crossing or template matching) and to define key search parameters. Search/Review controls **(Bottom)** are used for advancing forward/backward through the events, saving/rejecting detected events to/from the event table, setting the current search time (x=), activating auto event detection (All Waves) and managing the event table (here, the current table is “Event_C_Record_All_A”). Clicking the “review” checkbox turns off the search algorithm and allows users to inspect “successful” or “rejected” events, with the ability to move events between the two categories via the Save/Reject buttons. Once the user is satisfied with the event detection results, events can be copied to new waves via the “Events 2 Waves” button and the new waves can be selected for analysis (see Figure 9).

**Figure 9:**
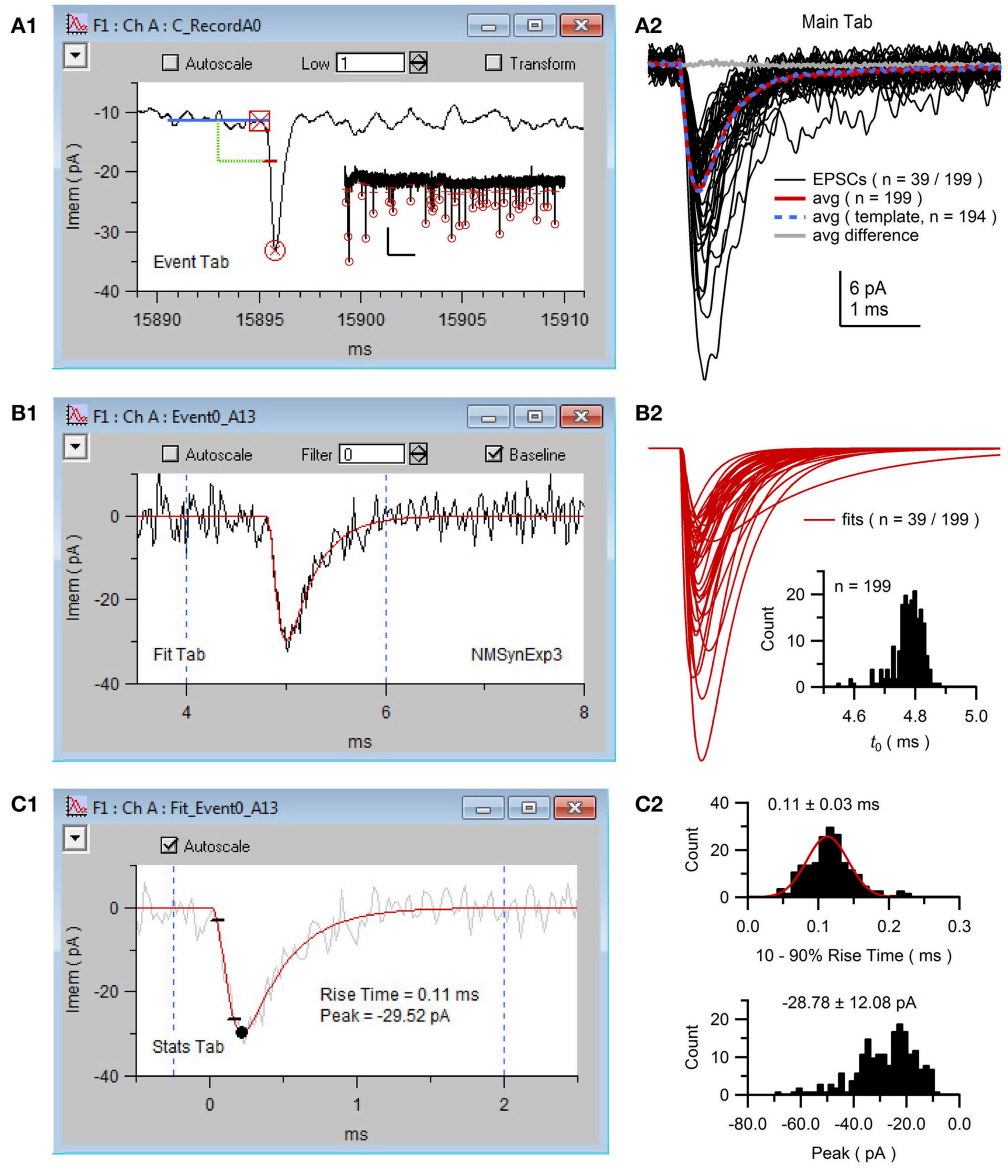
NeuroMatic acquisition, detection and analysis of spontaneous EPSCs. Spontaneous EPSCs with isolated AMPA receptor (AMPAR) component were recorded from the cell body of a GC. The stimulus protocol called Minis was configured via the Clamp tab to perform continuous whole-cell voltage-clamp recordings with 50 kHz sampling. **(A1)** Screen capture of the channel A graph during negative event threshold detection via the Event tab with criteria shown in Figure 8. Blue line denotes the sliding baseline window (5 ms). Green line denotes the negative threshold search criteria (2.5 ms ahead of the center of the baseline window, 6 pA below the baseline average). Red dash denotes the threshold crossing point of the detected EPSC. Red square and circle denote results of the EPSC onset and peak detection, respectively. Data (black line) was low-pass filtered at 1 kHz during event detection. *Inset*, first 39 of 199 detected EPSCs (thresholds and peaks) of the 170-s recording; x- and y-scale bars, 5 s and 20 pA. **(A2)** Detected EPSCs (*n* = 199) were copied to new waves (Event/Events 2 Waves) and selected for analysis via the *wave-prefix select* (“Event0_”). Black lines are the first 39 EPSCs overlaid (low-pass filtered at 5 kHz for display purposes). EPSCs were aligned to their onset (*t*_0_), as described in **(B2)**. Red line is the average of all 199 aligned unfiltered EPSCs. Event detection was repeated using this average for template matching (average normalized to have a baseline of 0 and peak of 1, truncated to 3 ms length with 0.2 ms baseline; detection criteria level = −3) resulting in 194 detected EPSCs whose average is plotted in blue. The difference between the two averages was near 0 (gray line). To run template matching, place NeuroMatic's MatchTemplate XOP (see http://www.neuromatic.thinkrandom.com/NMInstall.html) inside Igor's Extensions folder, activate “template matching” on the Event tab, select the template wave from the current data folder and click the Event tab's Match button. Scale bars are for data here and **(B2)**. **(B1)** Screen capture of the channel A graph during the curve fit (red line) of the same EPSC in **(A1)** to the multi-exponential function NMSynExp3 (Equation 1; second decay component was set to zero) via the Fit tab. EPSCs were fit between 4 and 6 ms (dashed blue lines), a window that contained the EPSC onset, peak and initial decay. Data was unfiltered during the fits. **(B2)** The first 39 of all 199 fits to the detected EPSCs. Fits were selected for analysis (wave prefix “Fit_Event0_”) and aligned on their onset (GUI/Main/X-scale/Align/align by a wave of values/FT_Syn3_X0_EV0All_A0). *Inset*, histogram of the onset (*t*_0_; see Equation 1) of all 199 fits (0.01 ms bins). **(C1)** Screen capture of the channel A graph during Stats 10–90% rise-time analysis of the curve fit in **(B1)** (red line). Black circle denotes the minimum peak value and small black lines denote times when the fit reached 10 and 90% of the peak value. Dashed blue lines denote window for minimum peak detection. The EPSC in **(B1)** was added to the background for display purposes (gray line). **(C2)** Histogram of 10–90% rise times (top; 0.01 ms bins; 2 EPSCs with slow rise times 0.5–0.7 ms are not shown) and minimum peaks (bottom; 2 pA bins) for the 199 fits computed via the Stats tab (Stats2/Functions/Histogram). The rise-time histogram was fit to a Gaussian function (red line; peak center at 0.11 ms, STDV = 0.03 ms; Menu/Analysis/Curve Fitting…/gauss). GC experimental details: cerebellar slice from a P25 rat, R_a_ = 15.5 MΩ, C_mem_ = 2.6 pF, V_hold_ = −80 mV. The external solution contained 10 mM MNI-glutamate. Data is from DiGregorio et al. (2007).

To search for spontaneous EPSCs, NeuroMatic's Event tab was activated and the “threshold < baseline” detection algorithm was selected for negative-deflecting events (Figure 8). This algorithm uses a sliding threshold search algorithm similar to that of Kudoh and Taguchi (2002), where a stepwise search begins at time zero for a data point that falls below a defined threshold level (Figure 9A1, red dash). The threshold level is a fixed value below baseline (e.g., 6 pA), but can also be the number of standard deviations (STDVs) below baseline (N_STDV_ < baseline). Here, “baseline” is a sliding average computed within a small window (blue line). Note, the sliding baseline window is an improvement to the sliding baseline point, *A*(*i*), used by Kudoh and Taguchi (2002) since the window, typically 2–10 ms, is less sensitive to fluctuating baseline noise. Once a potential event is registered by the threshold detector, there is an additional search for the event's onset (red square) and peak (red circle) within predefined windows. If either the onset or peak are not found, then the event is ignored and the search continues. However, for greater flexibility, NeuroMatic provides the ability to turn off the latter onset and/or peak detection criteria, which may be too restrictive. There is also a checkbox option to detect positive-deflecting events.

NeuroMatic event detection can be performed manually by clicking the “>” advance button (Figure 8). Once an event is detected, NeuroMatic displays the event in the channel graph as shown in Figure 9A1. The user then selects “Save” to save the event to a “successes” table or “Reject” to save the event to a “rejections” table. Alternatively, selecting “All Waves” activates NeuroMatic's automatic event detection, which searches for all events within all currently selected waves. For the use example here, “All Waves” was selected, after which a total of 213 events were detected, 5 of which were automatically rejected due to lack of a successful peak detection. Using the “review” option, where detected events are viewed sequentially in the active channel graph using the “<” and “>” buttons, another 14 events were manually rejected for having an unusual shape that did not match that of an EPSC. The remaining 199 events were copied to new waves with prefix “Event0” in the active data folder (Figure 9A2) via the “Events 2 Waves” button (events were copied 5 ms before and 25 ms after their threshold-crossing time). The new event waves were then selected for analysis via the *wave-prefix select* (Figure 2, control 2; “Event0”). Using the Fit tab, each event was curve fit with the following multi-exponential function (NMSynExp; Nielsen et al., 2004):

(1) $$\begin{array}{r} Y\left(t\right)={\left[1-e^{-\left(t-t_{0}\right)/\tau_{r}}\right]}^{n}\cdot \left[a_{d1}e^{-\left(t-t_{0}\right)/\tau_{d1}}+a_{d2}e^{-\left(t-t_{0}\right)/\tau_{d2}}\right] \end{array}$$

where *t* is time and the fit parameters are: *t*_0_ the onset time, τ_r_ the rise time constant, *n* the exponent, *a*_d1_ and τ_d1_ the amplitude and time constant of the fast decay component and *a*_d2_ and τ_d2_ the amplitude and time constant of the slow decay component (Figure 9B1). The resulting fit parameters were automatically saved to output waves and a table, and the curve fits automatically saved to waves in the active data folder. The fits were then selected for analysis via the *wave-prefix select* (“Fit_Event0_”) and, using the Main tab, aligned so their onset began at time zero (i.e., they were aligned to fit parameter *t*_0_; Figure 9B2). The minimum peaks and rise times of the fits were computed via the Stats tab (GUI/Stats/Stats1/RiseTime; Figures 9C1,C2). Computing statistics on the fits rather than the EPSCs reduces the effects of noise on these measures.

Besides the sliding threshold search algorithm, NeuroMatic can perform event detection using a template matching algorithm (Clements and Bekkers, 1997). In this algorithm, a waveform template with synaptic time course is stepwise “matched” to the data. This produces a “detection criterion” wave that is displayed in the active channel graph. An event is detected when the detection criterion wave crosses a predefined level (e.g., −3 or −4 for negative events). The waveform template can be a function such as an alpha or multi-exponential function, or a user-defined wave such as a normalized average EPSC. For comparison, we repeated the same analysis in Figure 9 using the template matching algorithm, where the template was the average of all 199 aligned EPSCs (Figure 9A2, red line) normalized between 0 and 1. In this case, there were 194 detected events, none of which had to be rejected. A total of 191 of these events were also detected by the sliding threshold search algorithm. Hence, for this particular GC, the two detection algorithms produced similar results (Figure 9A2, compare averages). However, the sliding threshold search algorithm required manual inspection/rejection of events with an unusual shape.

### Multiple-probability fluctuation analysis (MPFA) and simulation of quantal synaptic transmission

**Figure 10:**
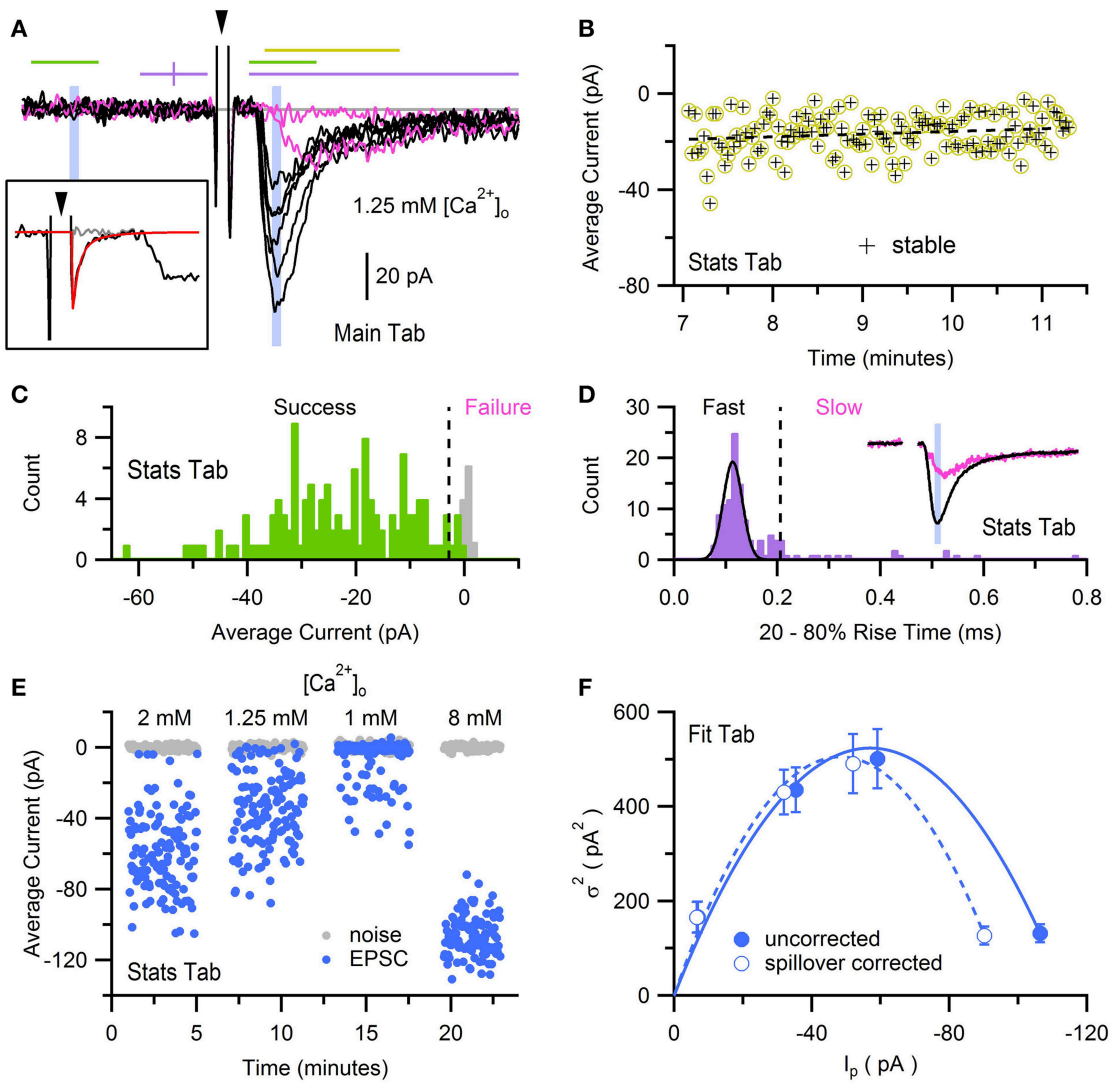
Multiple-probability fluctuation analysis with glutamate-spillover-current correction. Fluctuation analysis of AMPAR EPSCs recorded from a GC in whole-cell voltage-clamp mode in the presence of 1–8 mM [Ca^2+^]_o_, evoked by stimulation of a single MF input at 0.5 Hz. Data was acquired via an Axopatch 200B amplifier and pCLAMP software (100 kHz sample rate), saved in Axon binary format (ABF). **(A)** Eight superimposed EPSCs from a total of 128 evoked in ACSF containing 1.25 mM Ca^2+^. Black traces are fast-rising direct EPSCs and pink traces are non-direct EPSCs (one failure and one slow rising). Recordings were baseline subtracted (GUI/Main/Baseline) which computed the average current within a 1 ms window just before the stimulus (first purple line) and subtracted the average from the recording. The large biphasic current preceding the EPSC is the stimulus artifact (arrowhead, clipped at +27 pA) of which the final decay component was subtracted (see inset). Lines and shaded regions define Stats analysis windows as used in Sargent et al. (2005): stability (yellow, 2 ms), success/failure (green, 1 ms), rise time (purple, 5 ms, clipped to 4 ms), peak amplitude (blue, 0.1 ms). Background noise windows (green and shaded blue) are reflected at the baseline midline (purple vertical tick). Gray line denotes 0 current. *Inset*, a single stimulus artifact before (black) and after (gray) subtracting a double-exponential fit (red) to the final decay component of an average artifact computed from 16 recordings with no or little EPSC. **(B)** Stability analysis for the 128 EPSCs recorded in 1.25 mM Ca^2+^. Each data point denotes the average current in a 2 ms window that includes the EPSC peak, denoted by the yellow line in **(A)**. Dashed black line denotes regression analysis. For this data set the stable region included all 128 recordings. **(C)** Histograms (GUI/Stats/Stats2/Functions/Histogram; 1 pA bins) of average peak current (green) and background current (gray) of stable responses in **(B)** computed in the 1 ms windows denoted by green lines in **(A)**. Dashed line denotes 3 STDVs of the background current (−2.8 pA), the dividing line between failures and successes (*n* = 4 and 124, respectively). **(D)** Histogram (0.01 ms bins) of 20–80% rise times (see Figures 2, 3) of successful EPSCs defined in **(C)**, where baseline and peak detection windows are denoted by purple lines in **(A)**, and traces were low-pass filtered at 4 kHz. Black line is a Gaussian fit to the distribution (Menu/Analysis/Curve Fitting…/gauss). Dashed line at 0.22 ms denotes 5 STDVs above the Gaussian peak center, the dividing line between slow and fast EPSCs (*n* = 19 and 105, respectively). A second criteria based on the rise-time slope (GUI/Stats/Stats1/RTslope; not shown) re-categorized 7 slow EPSCs as fast. *Inset*, averages of EPSCs in the Fast set (black; *n* = 112) and Slow set (pink; *n* = 12). Blue shaded region is a 0.1 ms window centered on the peak of the average fast EPSC, the same window in **(A)**. **(E)** Average current of background noise (gray circles) and EPSC peak (blue circles) of stable responses in **(B)**, computed in blue shaded 0.1 ms windows in **(A)**. Analysis was repeated for recordings in 1, 2 and 8 mM [Ca^2+^]_o_. **(F)** $\sigma_{I}^{2}-I_{p}$ relation computed from data in panel **(E)** before and after spillover correction (closed and open circles, respectively). Lines are multinomial fits (Equation 2) with values given in the main text. GC experimental details: cerebellar slice from a P25 rat, R_a_ = 28 MΩ, C_mem_ = 3.4 pF, V_hold_ = −76 mV. Data is from Sargent et al. (2005), their Figure 3.

Here, we use previously published data of AMPA receptor (AMPAR) EPSCs recorded from a mossy-fiber (MF)-GC synapse to determine the synaptic quantal parameters using MPFA with glutamate-spillover-current correction (Sargent et al., 2005). The EPSCs were evoked by stimulation of a single MF input at 0.5 Hz and contained a fast component mediated by direct release of glutamate from the synapse, and a slow component mediated by glutamate spillover from neighboring synapses (Figure 10A; DiGregorio et al., 2002; Nielsen et al., 2004). To perform MPFA (Silver, 2003), the recordings were imported into a NeuroMatic data folder via the Open Data Files option (Figure 2, control 1) and baseline subtracted using a 1 ms window just before the stimulus artifact (GUI/Main/Baseline). To remove contamination of the stimulus artifact from the onset of the EPSCs, an average artifact was computed from those recordings where the stimulus did not evoke an EPSC (computed using the Stats and Main tab), a double exponential was fit to the final decay of the average artifact (Fit tab), and the resulting fit was subtracted from the individual recordings (GUI/Main/Scale/scale by a wave point-by-point; Figure 10A, inset). A stable period of consecutive post-synaptic responses was identified using the Spearman rank-order correlation test (Figure 10B; GUI/Stats/Stats2/Functions/Stability). NeuroMatic's Stability function placed the stable responses in a set called “stable,” which was then selected for further analysis via the *wave/set select* (Figure 2, control 6). Stable responses were subdivided into either a “Success” or “Failure” set using a Stats amplitude criterion: a response was categorized as Success if its average current over a 1 ms window following the stimulus was >3 STDVs of the average background current; otherwise, the response was categorized as Failure (GUI/Stats/Stats2/Functions/Inequality; Figure 10C). EPSCs in the Success set were then separated into “Fast” and “Slow” sets using a Stats rise-time criteria: an EPSC was categorized as Slow if its rise time was greater than the peak center plus 5 STDVs of a Gaussian fit to the rise-time distribution; otherwise, the EPSC was categorized as Fast (Figure 10D). Because this procedure results in some EPSCs with small fast-rising components in the Slow set (DiGregorio et al., 2002), EPSCs in the Slow set were further separated into a “Fast2” and “Slow2” set using a Stats rise-time slope (RTslope) criteria: an EPSC was categorized as Fast2 if its rise-time slope was >65 pA/ms, otherwise it was categorized as Slow2. A final “AllFast” set was created (GUI/Sets/New/“AllFast”) and populated with the EPSCs in the Fast and Fast2 sets (GUI/Sets/Equation/AllFast = Fast OR Fast2). Examples of fast EPSCs are shown in Figure 10A, as well as a failure and slow spillover current. To determine the amplitude time window to compute MPFA, EPSCs in the AllFast set were averaged (GUI/Main/Average) and a 0.1 ms Stats window was centered on the peak of the average using the Stats1 Min function (Figure 10D, inset). For the background noise window, the 0.1 ms window was reflected in time about the midpoint of the baseline window (Figure 10A). The peak current and baseline noise were then measured for all responses (i.e., successes, slow spillover currents and failures) in the stable set (Figure 10E; GUI/Stats/Stats1/Avg), and results of these measures were used to compute the mean peak current (*I*_p_), variance ($\sigma_{I}^{2}$) and error of the variance derived from *h*-statistics (GUI/Stats/Stats2/Functions/MPFA Stats; Saviane and Silver, 2006a). The above analysis was repeated for 3 more data sets acquired under different extracellular Ca concentrations ([Ca^2+^]_o_; Figure 10E) and a $\sigma_{I}^{2}-I_{p}$ relation was created using all 4 conditions of [Ca^2+^]_o_ (Figure 10F). The $\sigma_{I}^{2}-I_{p}$ relation was fit with the following multinomial equation (Menu/Analysis/Curve Fitting…/NM_MPFA1):

(2) $$\begin{array}{r} \sigma_{I}^{2}=\left[Q_{p}I_{p}-I_{p}^{2}/N\right]\left[1+CV_{QII}^{2}\right]+Q_{p}I_{p}CV_{QI}^{2} \end{array}$$

where *N* is the number of functional release sites, *Q*_*p*_ is the quantal amplitude measured at the peak, *CV*_*QI*_ is the coefficient of variation of the quantal size at an individual site (due to amplitude and latency variability) and *CV*_*QII*_ is the coefficient of variation of the quantal size across sites (Silver, 2003). During the fit, *CV*_*QI*_ and *CV*_*QII*_ were fixed to predetermined values (0.39 and 0.31, respectively; Sargent et al., 2005) and the errors of the variance were used as weights. Results of the fit produced *N* = 6.8 ± 0.6 and *Q*_*p*_ = −14.7 ± 1.2 pA. To remove the effects of the spillover current, which tends to increase *N* and decrease *Q*_*p*_, *I*_p_ and $\sigma_{I}^{2}$ of the spillover current were estimated from relations derived from experimental data (Sargent et al., 2005; their Figure 3D) and subtracted from *I*_p_ and $\sigma_{I}^{2}$ for all responses (Figure 10F). Fitting Equation (2) to the spillover corrected data produced *N* = 5.1 ± 0.5 and *Q*_*p*_ = −16.7 ± 1.4 pA.

**Figure 11:**
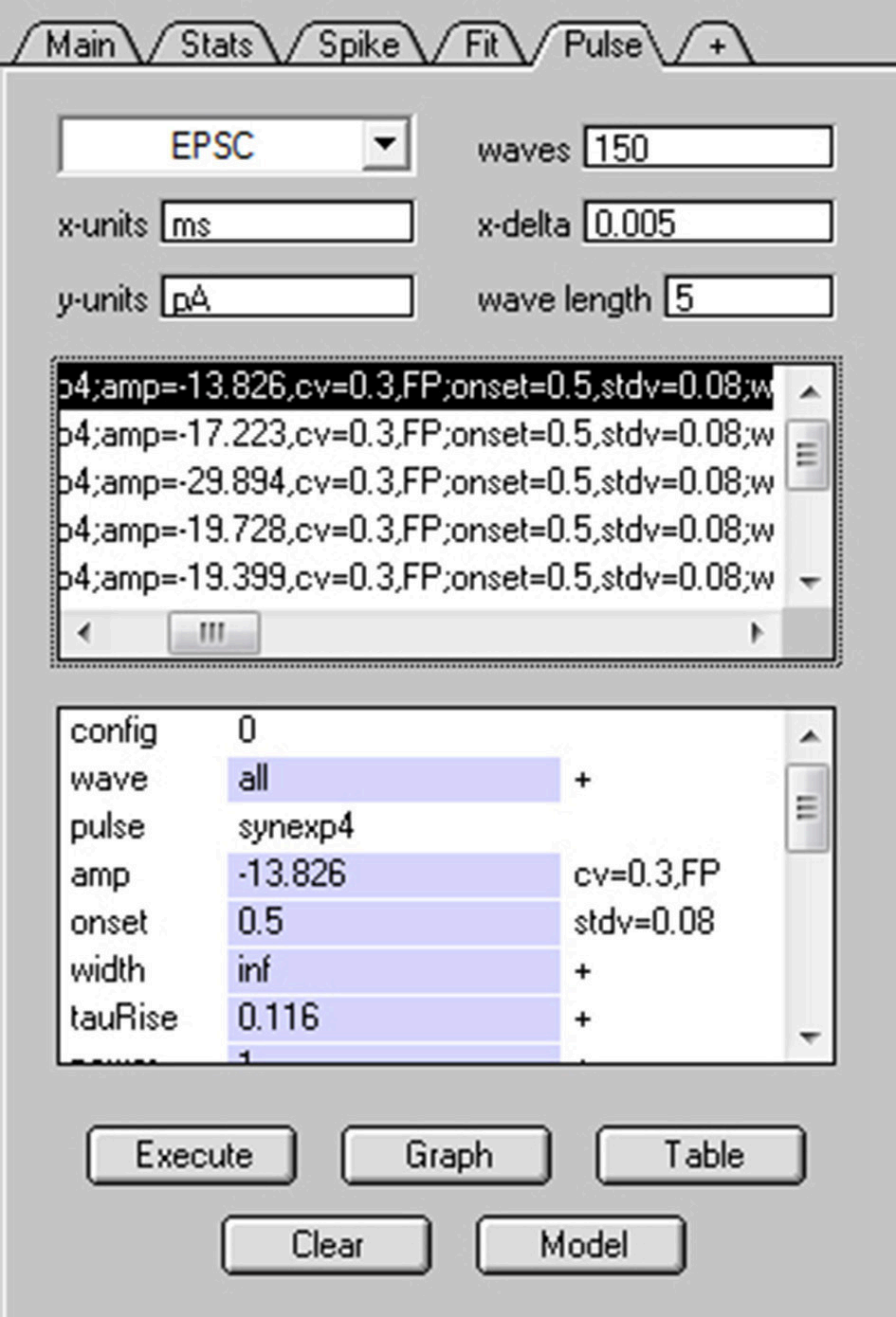
Simulating stochastic synaptic release via NeuroMatic's Pulse tab. Screen capture of NeuroMatic's Pulse tab after clicking the Model button, selecting “Granule Cell Multinomial Synapse” and entering the following parameters via NeuroMatic's prompts: *N* = 5, *Q*_*p*_ = −20 pA, *P* = 0.5, *CV*_*QS*_ = 0.3, σ_*QL*_ = 0.08 ms, *CV*_*QII*_ = 0.3, no spillover. The time course of quantal release at each site was automatically set to the NeuroMatic function NMSynExp (Equation 1) with values: τ_r_ = 0.116 ms, *n* = 1, *a*_d1_ = 86.72, τ_d1_ = 0.36 ms, *a*_d2_ = 13.28, τ_d2_ = 2.034 ms. Final configurations for each release site were listed on the Pulse tab, as shown here, including each site's *P* (binomial = 0.5). After clicking the Execute button, NeuroMatic generated 150 output waves with prefix name “EPSC.” The waves were automatically selected for visualization and analysis (see Figure 12).

**Figure 12:**
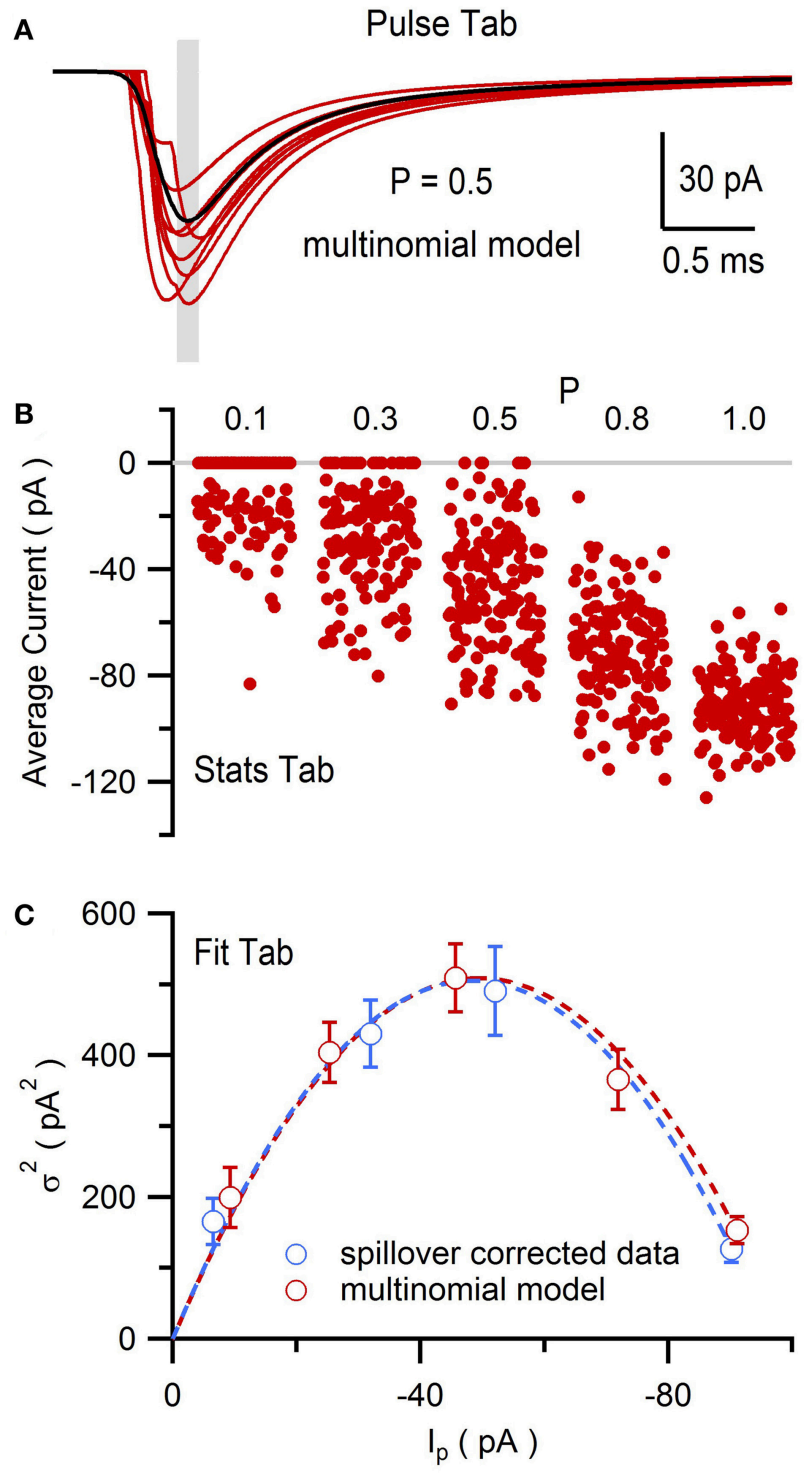
Multiple-probability fluctuation analysis of simulated EPSCs generated by NeuroMatic's Pulse tab. Multinomial model of a MF-GC synapse as described in Figure 11. **(A)** Eight superimposed simulated synaptic AMPAR currents (red) from a total of 150. Gray shaded region denotes the 0.1 ms Stats window centered on the peak of the average of all 150 traces (black) used to compute the peak amplitude (*I*_p_). *P* = 0.5 for each site. **(B)** *I*_p_ for *P* = 0.1, 0.3, 0.5, 0.8 and 1.0. There were 150 trials for each *P*. **(C)** $\sigma_{I}^{2}-I_{p}$ relation (red circles) computed from data in **(B)** with its fit to Equation (2) (red line; *N* = 5.3 ± 0.4 and *Q*_*p*_ = −16.8 ± 1.1 pA, *CV*_*QI*_ ≡ 0.36, *CV*_*QII*_ ≡ 0.30, where ≡ denotes fixed fit values). Spillover-corrected experimental data (blue circles) and fit (blue line) are from Figure 10F for comparison.

Using NeuroMatic's Pulse tab (Figure 11) a simple Monte Carlo model was used to simulate these experimental results (Sargent et al., 2005; Saviane and Silver, 2006a; Minneci et al., 2012). The model was configured by clicking the Pulse tab's Model button, selecting “Granule Cell Multinomial Synapse” and setting the number of release sites (*N*) to 5, quantal peak amplitude (*Q*_*p*_) to −20 pA, probability of release (*P*) to 0.5 and number of trials to 150. Within-site variability (*CV*_*QI*_) was included by setting the coefficient of variation of *Q*_*p*_ (*CV*_*QS*_) to 0.3 and the standard deviation of the latency/onset (σ_QL_) to 0.08 ms (producing *CV*_*QL*_ = 0.2; Saviane and Silver, 2006a). Together, these within-site variance contributions resulted in *CV*_*QI*_ = 0.36 ($CV_{QI}^{2}=CV_{QS}^{2}+CV_{QL}^{2}$). The across-site coefficient of variation of *Q*_*p*_ (*CV*_*QII*_) was set to 0.3. The option to fix *CV*_*QII*_ to a given precision was chosen; in this case, NeuroMatic iteratively created sets of 5 *Q*_*p*_ until the set's mean and variability approximated the desired values with the entered precision of 1%. The time course of quantal events was simulated by Equation (1) using kinetics and amplitudes representative of excitatory miniature currents. To simulate only the fast direct component, the option to add spillover current was not chosen. Final configuration parameters for the 5 release sites were listed in the Pulse tab's configuration window for viewing and editing (Figure 11). The simulation was then run by clicking the Pulse tab's Execute button. On each trial, NeuroMatic determined whether release occurred at an individual release site by generating a random number within the interval [0,1] and comparing it to the site's *P*. If the number was smaller than *P*, then release occurred at the site and contributed to the total synaptic current. For each successful release event, within-site variability of *Q*_*p*_ and latency were added by sampling from their appropriate Gaussian distribution. Successful releases from the 5 sites were then summed and saved in a wave with prefix name “EPSC” followed by the trial number. A total of 150 trial waves were computed (Figure 12A) which were automatically selected for visualization and analysis. To compute MPFA, the above simulation was repeated for *P* = 0.1, 0.3, 0.8 and 1.0 (Figure 12B) and a $\sigma_{I}^{2}-I_{p}$ relation was created using all 5 conditions of *P* (Figure 12C). The final $\sigma_{I}^{2}-I_{p}$ relation was similar to that of the experimental data with spillover-current correction.

### Short-term plasticity of synaptic conductances: acquisition, analysis and simulation

**Figure 13:**
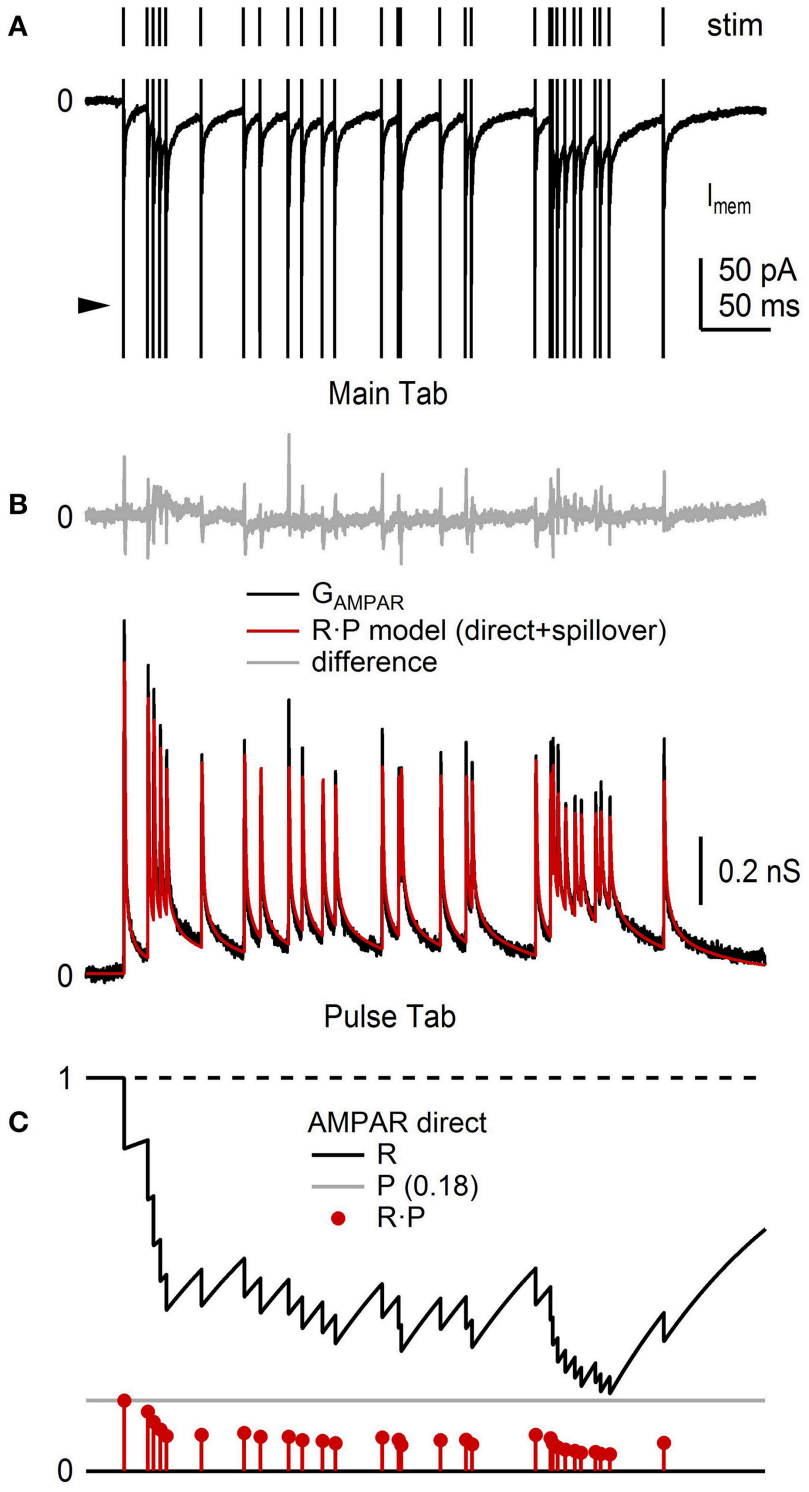
NeuroMatic analysis and simulation of synaptic short-term plasticity. Analysis of short-term depression of EPSCs recorded from the cell body of 4 GCs. Data was recorded in voltage-clamp mode via an Axopatch 200B amplifier and Axograph software (100 kHz sample rate). **(A)** Train of GC EPSCs containing an AMPAR and NMDAR component (bottom; control; average of 10 repetitions) evoked by stimulation of a single MF input at random Poisson intervals with mean rate of 85.6 Hz (top). The large fast currents before each EPSC are stimulus artifacts (arrowhead) which have been truncated for display purposes. The NMDAR component was isolated by taking subsequent recordings in 5 mM NBQX (not shown). **(B)** Average G_AMPAR_ train (black) computed from EPSC trains, such as the one in **(A)**, from 4 GCs, as described in the main text. *Red trace* is an *R*·*P* plasticity model computed via the Pulse tab (Figure 11) consisting of a direct and spillover component each having independent *R* and *P* dynamic variables. Top: difference between the data and model (gray). The brief transients (<1 ms) are due to small mismatches at the peak of the direct components. Model parameters were from Billings et al. (2014), with adjustments to the amplitude and *P*_inf_ values to simulate this particular G_AMPAR_ train. **(C)** Plasticity model parameters *R* (black), *P* (gray) and *R*·*P* (red circles) of the direct component of the G_AMPAR_ train in **(B)**, where *R*_inf_ = 1, *P*_inf_ = 0.14, Δ = 0, τ_R_ = 131 ms. Parameters for the spillover component (not shown) were *R*_inf_ = 1, *P*_inf_ = 0.68, Δ = 0, τ_R_ = 14.85 ms. GC experimental details: cerebellar slices from P40 rats, R_a_ = 17–24 MΩ, C_mem_ = 2.4–6.0 pF, V_hold_ = −60 or −66.3 mV. Data is from Rothman et al. (2009).

In this final example, we use NeuroMatic acquisition, analysis and simulation to investigate short-term plasticity at the MF-GC synapse which is known to have a pre-synaptic component (facilitation and depression of *P*) and post-synaptic component (depression of *Q*_*p*_; Saviane and Silver, 2006b). First, previously published recordings of GC EPSCs evoked at random intervals by stimulation of a single MF input (Figure 13A; Rothman et al., 2009) were imported into a NeuroMatic data folder via the Open Data Files option (Figure 2, control 1). The EPSC trains, which contained AMPAR and NMDA receptor (NMDAR) components, were converted to conductance trains by (1) subtracting the baseline current (GUI/Main/Baseline), (2) removing the stimulus artifacts (GUI/Main/Operations/Clip Events), (3) averaging 10 responses to the same MF stimulation train (GUI/Main/Average), and (4) dividing by the driving force after correcting it for the electrode junction potential (GUI/Main/Scale/scale by a value). This analysis was repeated for 4 GCs, the results of which were averaged together by (1) aligning the conductance trains at their 20% rise-time point of the first synaptic response (Stats 20–80% RiseTime; GUI/Main/X-scale/Align/align by a wave of values), (2) interpolating the 4 trains to the same x-scale (GUI/Main/X-scale/Interpolate), and (3) averaging the 4 trains (GUI/Main/Average). To isolate the AMPAR and NMDAR components, the above analysis was repeated for EPSC trains recorded in the presence of 5 mM NBQX, from the same 4 GCs (not shown). The resulting conductance trains, containing only the NMDAR component (G_NMDAR_), were subtracted from the conductance trains recorded under control conditions, resulting in conductance trains containing only the AMPAR component (G_AMPAR_). The G_AMPAR_ trains contained a fast direct and slow glutamate-spillover component, the former of which showed clear signs of synaptic depression at high stimulus frequencies (Figure 13B).

Next, using the Pulse tab (Figure 11), synaptic depression of the G_AMPAR_ trains was simulated using a plasticity model containing two dynamic variables, one representing a resource pool *R*, the other the pool's release probability *P* (Figures 13B,C; Varela et al., 1997; Tsodyks et al., 1998; Billings et al., 2014; Rothman and Silver, 2014). In this model, the maximum synaptic response (G_max_) is scaled by the product *R*·*P* at a given stimulus time, after which *R* is decremented to reflect a loss of resource (here, representing both pre- and post-synaptic depression) and *P* is incremented to reflect an increase in the vesicle-release probability due to a rise in internal [Ca^2+^] (i.e., pre-synaptic facilitation):

(3) $$\begin{array}{r} R\to R-R\cdot P\;P\to P+\Delta \left(1-P\right) \end{array}$$

where Δ is a scale factor between 0 and 1. After adjusting *R* and *P*, the two variables follow an exponential time course back to their initial values *R*_inf_ and *P*_inf_ with time constants τ_R_ and τ_P_, described by the following differential equations:

(4) $$\begin{array}{r} \frac{dR}{dt}=\frac{\left(R_{inf}-R\right)}{\tau_{R}}\;dP/dt=\frac{\left(P_{inf}-P\right)}{\tau_{P}} \end{array}$$

Because the G_AMPAR_ trains had direct and spillover components, the trains were simulated by summing two independent plasticity models with their own *R* and *P*. Moreover, because the direct and spillover components showed no obvious signs of facilitation, Δ was set to 0 in both models. The time course of G_max_ for the direct and spillover conductance waveforms was described by an exponential function containing multiple rise and decay components whose parameters were taken from Billings et al. (2014; their Table S3). Comparison of the simulated and experimental G_AMPAR_ trains showed a close agreement (Figure 13B), which has previously been shown for a similar plasticity model over a range of MF input frequencies (Schwartz et al., 2012; *D*·*F* model; <90 Hz MF input). Using the same *R*·*P* model, synaptic plasticity of the corresponding G_NMDAR_ trains was also simulated, but this time with facilitation (not shown, but see Schwartz et al., 2012, their Figure 6).

**Figure 14:**
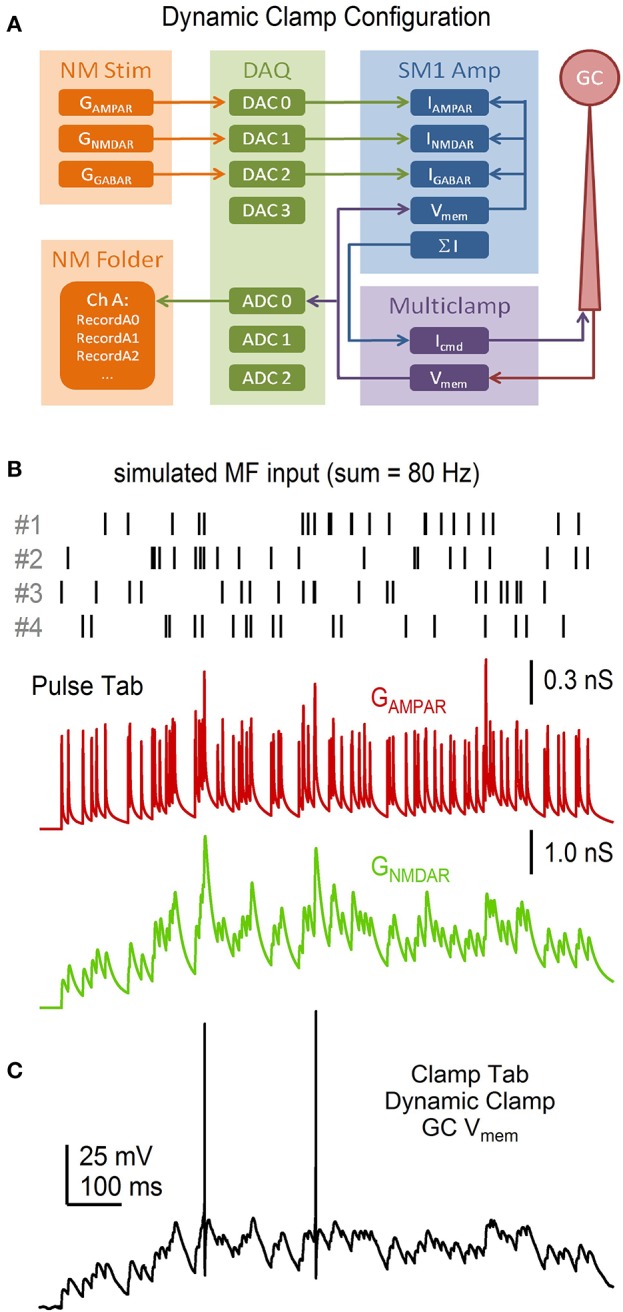
Dynamic-clamp acquisition using simulated synaptic conductance trains. NeuroMatic Pulse tab *R*·*P* plasticity models were used to simulate 4 statistically different MF inputs with mean spike rate *f*. The 4 MF inputs were summed together and injected into a real GC via dynamic clamp. **(A)** Flowchart of the dynamic-clamp experiment. NeuroMatic sent precomputed G_AMPAR_, G_NMDAR_ and G_GABAR_ waves to 3 DAC channels of a DAQ device, which were connected to the appropriate input channels of a dynamic-clamp SM1 amplifier. In real time, the SM1 amplifier converted the conductances to currents using V_mem_ of the GC, via the Multiclamp amplifier, and preset reversal potentials, i.e., I = G·(V_mem_ – V_rev_). The sum of the 3 currents was sent to the command input of the Multiclamp amplifier (I_cmd_). The Multiclamp amplifier recorded the GC V_mem_ response to I_cmd_ and sent V_mem_ to an ADC channel of the DAQ device, which was read by NeuroMatic. **(B)** Synaptic event times for 4 simulated MF inputs (top) computed by the Pulse tab. Event times had random Poisson intervals >1 ms with *f* = 20 Hz. The G_AMPAR_ train (middle) was computed from the 4 event trains, where each train had its own *R*·*P* plasticity model as described in Figure 13, and the resulting 4 conductance trains were summed together. The G_NMDAR_ train (bottom; before Mg^2+^ block) was computed in the same way, but included facilitation. Parameters for the G_AMPAR_ and G_NMDAR_ trains are as reported in Billings et al. (2014). **(C)** GC V_mem_ in response to injection of the G_AMPAR_ and G_NMDAR_ trains in **(B)** via dynamic clamp. The injection included a tonic G_GABAR_ of 0.27 nS. Reversal potentials of the AMPAR, NMDAR and GABAR channels were 0, 0 and −75 mV, respectively. The resting potential of this recording was −75 mV. GC experimental details: cerebellar slice from P24 rat, R_a_ = 26.7 MΩ, C_mem_ = 2.6 pF.

As the simultaneous independent stimulation of each MF input to a GC is not feasible (there are ~4 inputs), we used the above *R*·*P* plasticity models to study the input-output relation of a GC by simulating 4 different MF inputs and injecting them into the GC via a conductance-clamp amplifier (Figure 14A; Robinson and Kawai, 1993). To do this, the Pulse tab's Model button was clicked and “Granule Cell Synaptic Conductance Train with STD” was selected. The number of “input trains per wave” was set to 4 (default option). NeuroMatic then created 4 sets of Pulse configurations, one for each MF input, each with a different train of random MF input times (Figure 14B). After clicking the Pulse tab's Execute button, NeuroMatic fed the 4 trains of MF input times into the *R*·*P* plasticity models and summed the 4 inputs. A total of 7 different G_AMPAR_ and G_NMDAR_ trains were simulated for the following MF input frequencies: 10, 20, 30, 50, 70, 90 and 120 Hz. In order to readily use the G_AMPAR_ and G_NMDAR_ trains for dynamic clamp, “MyDAC_0_” and “MyDAC_2_” were entered as the output wave prefix name on the Pulse tab (corresponding to the DAC configurations #0 and 2, respectively) and the Pulse tab sample rate was set to match that of the Clamp stimulus protocol (16.67 kHz). Using the Main tab Copy function, the G_AMPAR_ and G_NMDAR_ waveforms were copied to a Clamp stimulus folder called GtrainFF and the “use ‘My’ waves” option was activated on the Clamp/Stim/Pulse tab. To simulate a tonic GABA receptor conductance (G_GABAR_), waves with the prefix name “MyDAC_1_” and a 16.67 kHz sample rate were created (GUI/Main/Edit/Make), set equal to a constant conductance value (GUI/Main/Scale/scale by a value/“ = ” 0.27) and copied to the same stim folder (GUI/Main/Copy). After gaining whole-cell access to a GC in current-clamp mode, protocol GtrainFF was activated on the Clamp tab via the *stimulus select* (Figure 6, control 1) and the Clamp tab's Record button was clicked. Using the predefined output channel numbers of the Clamp DAC configurations #0-2, NeuroMatic sent the G_AMPAR_, G_NMDAR_ and G_GABAR_ waveforms to the appropriate ITC DAC outputs which were connected to a 3-channel dynamic-clamp SM1 amplifier (Cambridge Conductance; Figure 14A). NeuroMatic recorded the GC membrane response to current injection from the SM1 amplifier (Figure 14C) and saved the results in a NeuroMatic data folder, which was saved to a hard disk once acquisition finished. Such dynamic-clamp experiments have previously been used to study the effects of short-term depression on gain modulation at the MF-GC synapse (Rothman et al., 2009).

**Figure 15:**
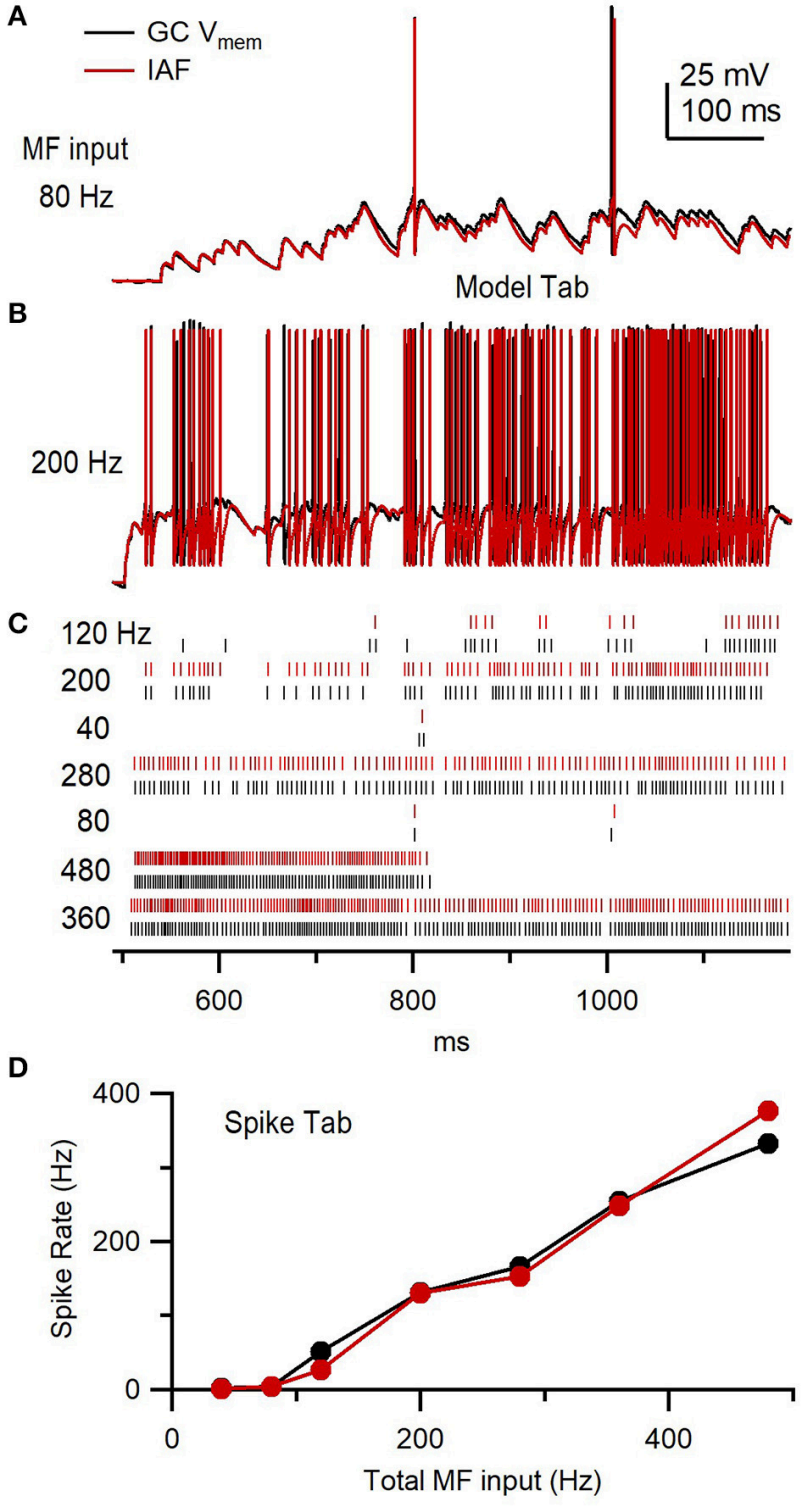
Granule cell versus integrate-and-fire model response to the same simulated synaptic conductance trains. **(A)** GC membrane potential (V_mem_) from Figure 14C (black) compared to an IAF model response (red) to injection of the same G_AMPAR_ and G_NMDAR_ waveforms (80 Hz total MF input rate) and tonic G_GABAR_. The IAF response was computed using NeuroMatic's Model tab as described in Supplementary Figure 6. **(B)** Same as **(A)** but for G_AMPAR_ and G_NMDAR_ computed with 4 simulated MF inputs with *f* = 50 Hz (i.e., 200 Hz total MF input rate) via the Pulse tab. **(C)** Spike raster plots computed from V_mem_ responses in **(A,B)**, and 5 other responses for different *f*, using the Spike tab. The total MF input rate is denoted on the left. **(D)** Output spike rate versus total MF input rate using data in **(C)**.

Finally, we used NeuroMatic's Model tab (Supplementary Figure 6) to tune a simple IAF model so that its firing properties matched those of the GC shown in Figure 14C. To do this, the same G_AMPAR_, G_NMDAR_ and G_GABAR_ trains used in the dynamic-clamp experiment were “injected” into the IAF model and the model's membrane parameters (capacitance and resistance) and action-potential (AP) parameters (threshold, peak, reset potential, refractory period) were adjusted until the model replicated the experimental data with respect to the membrane potential (Figures 15A,B), spike raster plots (Figure 15C) and input-output spike frequency relations (Figure 15D). Such an IAF model has previously been used to study the effects of incomplete Mg^2+^ block of NMDARs on synaptic transmission at the MF-GC synapse (Schwartz et al., 2012).

## Discussion

Here, we described NeuroMatic version 3.0, a freely available software toolkit that integrates acquisition, analysis and simulation of electrophysiological data into a single package with an easy-to-use GUI. With our four use examples, we demonstrated a wide range of functionality, including multiple modes of data acquisition (voltage clamp, current clamp, episodic, continuous, triggered stimulation, and dynamic clamp via a dedicated analog signal processing board), multiple types of data analysis (spike detection, spontaneous event detection, curve fitting, MPFA, artifact subtraction, wave alignment, stability analysis) and simulations of neural activity (stochastic synaptic transmission, synaptic short-term plasticity, IAF models). Moreover, with each use example, we demonstrated the tight integration of NeuroMatic's multiple toolkit components that creates an efficient workflow for a variety of experimental paradigms including those requiring a recursive element (Figure 1). Finally, by providing our own user-defined functions for computing the data analysis and simulations and generating the figures for each use example (see Supplementary Material), we demonstrated how straightforward it is to generate customized functions using NeuroMatic and Igor commands. The user-defined functions, along with Igor's Command Window and wave notes, also provide a model for creating open-access communications with transparent and reproducible workflows, starting from data acquisition and ending in the generation of graphs and figures (Eglen et al., 2017; Munafò et al., 2017).

Besides NeuroMatic, there are a few other open-source software packages that provide a broad range of functionality for neurophysiology. ACQ4 (Campagnola et al., 2014) is an open-source cross-platform package written in Python that integrates data acquisition and analysis. ACQ4 is particularly useful for experimentalists who need to combine electrophysiology, photo-stimulation and imaging (multiphoton, calcium or intrinsic imaging) since the package comes with a built-in device manager capable of controlling/synchronizing several types of hardware, including DAQ boards, cameras, lasers, shutters, scan mirrors and motorized stages. While ACQ4 does provide analysis modules for event detection and current-voltage relations, most of its analysis modules are related to imaging. RELACS (http://relacs.sourceforge.net) is an open-source Linux-platform package written in C++ that specializes in acquisition of electrophysiological data, and comes with a library of basic data analysis routines, including statistics, curve fitting and event detection. WinWCP (http://spider.science.strath.ac.uk/sipbs/software_ses.htm) is an open-source Windows-platform package written in Delphi/Pascal that has similar functionality to RELACS, but also performs non-stationary noise analysis. Ephus (Suter et al., 2010) is an open-source Windows-platform package written in MATLAB that specializes in data acquisition. Like ACQ4, Ephus is useful for experimentalists who need to combine electrophysiology and photo-stimulation. Although broad in scope, these software packages have been written with an emphasis on data acquisition; data analysis has been added to the software with limited scope, in which case users need to code their own analysis routines, or port their data to other software packages. One package that does provide a broad range of data analysis routines is Stimfit (Guzman et al., 2014), an open-source cross-platform package written in C++ with a Python scripting interface. Stimfit, however, does not perform data acquisition. Other open-source packages like Spyke Viewer (Python; Pröpper and Obermayer, 2013), Elephant (Python; http://neuralensemble.org/elephant/) and sigTOOL (MATLAB; Lidierth, 2009) focus on providing tools for spike train analysis similar to those provided on NeuroMatic's Spike tab, while other packages like Chronux (MATLAB; Bokil et al., 2010), nSTAT (MATLAB; Cajigas et al., 2012), Tridesclous (Python; https://github.com/tridesclous/tridesclous) and PRANAS (C++; Cessac et al., 2017) focus on providing more specialized tools for signal analysis, spike sorting and multi-electrode-array data. These latter spike analysis tools provide functionality that is currently outside the scope of NeuroMatic. In comparison, however, NeuroMatic provides a broader range of basic built-in functionality for data acquisition and analysis, and although designed for electrophysiology, can be readily used in conjunction with photo-stimulation and imaging experiments (Supplementary Figure 7).

For simulations, NeuroMatic's Pulse and Model tabs are useful for developing and testing models of stochastic synaptic release, synaptic plasticity and membrane conductances, since the models can be readily compared to real data and model parameters are easily accessible for adjustment. Neuromatic's Pulse tab is also useful for generating customized patch-clamp commands such as synaptic conductance waveforms for dynamic clamp. However, because the Model tab only performs single-compartment IAF and Hodgkin-Huxley-like simulations, those needing to perform multi-compartment or neural network simulations should use packages like Neuron (Hines and Carnevale, 1997), Genesis (Bower and Beeman, 2007), Moose (https://github.com/BhallaLab/moose), NEST (http://www.nest-simulator.org) or Brian (Goodman and Brette, 2009). These packages are also significantly faster than NeuroMatic's Model tab.

Future plans for development of NeuroMatic include regions of interest (ROI) image analysis (Fernández-Alfonso et al., 2014). While NeuroMatic already supports reading and writing data in the popular industry-standard HDF5 format, we plan to extend NeuroMatic's input/output functionality to support reading and writing in the Neurodata Without Borders (NWB) format (Teeters et al., 2015), a new standardized format (based on HDF5) designed to store a variety of neurophysiology data. In the longer term, if NWB becomes widely used, NeuroMatic could be restructured to directly use the NWB format as its internal data folder structure. The adoption of more standardized formats for data and metadata will allow integration with data sharing projects and promote transparent, open and reproducible neuroscience research (Nosek et al., 2015; Ascoli et al., 2017; Eglen et al., 2017; Gleeson et al., 2017; Munafò et al., 2017).

One caveat of using NeuroMatic is that it requires the purchase of Igor, and for those needing to acquire data from a NI-DAQ device requires the purchase of Igor's NI-DAQ Tools MX XOP. However, the current cost of Igor including XOP) is ~2–6 times less than that of acquisition packages like AxoGraph, Spike2 (Cambridge Electronic Design; http://ced.co.uk), HEKA Patchmaster and pCLAMP (costs compared for a single-seat license in an academic setting). Hence, NeuroMatic in conjunction with Igor can provide a cost-effective means of providing acquisition, analysis and simulation for electrophysiologists in a single package.

## Materials and methods

All animal experiments were conducted in accordance with the Animals Scientific Procedures Act of 1986, United Kingdom Home Office, and approved by the UCL ethics review board. For the data in Figures 3, 7, 14, parasagittal slices (220–225 μm) of the cerebellar vermis were prepared from wild-type mice or rats (see legends) in artificial cerebrospinal fluid (ACSF) containing (in mM): 125 NaCl, 2.5 KCl, 2 CaCl_2_, 1 MgCl_2_, 1.25 NaH_2_PO_4_, 26 NaHCO_3_, 25 glucose, 0.5 ascorbic acid, or in a low-sodium sucrose solution containing: 2.5 KCl, 0.5 CaCl_2_, 4 MgCl_2_, 1.25 NaH_2_PO_4_, 24 NaHCO_3_, 25 glucose, 230 sucrose, 0.5 ascorbic acid. Slices were incubated in ACSF at ~32°C for ~30 min, then allowed to cool to ~21°C. During whole-cell patch-clamp recordings, slices were bathed in ACSF maintained at ~36°C. All extracellular solutions were bubbled with 95% O_2_, 5% CO_2_. For Figure 3, EPSCs were recorded from a GC in whole-cell voltage-clamp mode using an ITC-18 DAQ board (InstruTECH/HEKA; 50 kHz sampling), an Axopatch 200B amplifier (10 kHz filter) and micropipettes containing (in mM): 110 MeSO_3_, 4 NaCl, 1.78 CaCl_2_, 0.3 Na-GTP, 4 Mg-ATP, 40 HEPES and 5 EGTA (pH adjusted to 7.3 with KOH). The ACSF contained 10 μM AP5, 20 μM 7-chlorokynurenic acid, 10 μM SR 95531 and 0.3 μM strychnine to isolate the AMPAR-mediated current. A single MF input was evoked by a second pipette positioned in the GC layer. For Figure 7, the membrane potential of a GC was recorded in whole-cell current-clamp mode using an ITC-18 DAQ board (50 kHz sampling), an Axopatch 200B amplifier (10 kHz filter) and micropipettes containing (in mM): 114 MeSO_3_, 6 NaOH, 3 MgCl_2_, 0.02 CaCl_2_, 0.3 Na-GTP, 4 Na-ATP, 40 HEPES and 0.15 BAPTA (pH adjusted to 7.3 with KOH). For Figure 14, the membrane potential of a GC was recorded in whole-cell current-clamp mode using an ITC-18 DAQ board (16.67 kHz sampling), a Multiclamp 700B amplifier (Molecular Devices; 7 kHz filter) and micropipettes containing (in mM): 110 KMeSO_4_, 6 NaOH, 3 MgCl_2_, 0.02 CaCl_2_, 0.3 Na-GTP, 4 Na-ATP, 40 HEPES and 0.15 BAPTA (pH 7.3). For the data in Figures 9, 10, 13, methods can be found in the reference cited at the end of each legend. All reported holding potentials (V_hold_) have been corrected for the liquid junction potential.

## Author contributions

JR is the primary developer of NeuroMatic and wrote the first draft of the manuscript. RAS contributed to the conceptual design of NeuroMatic and to planning and writing the manuscript.

### Conflict of interest statement

JR received funding from WaveMetrics in 2001–2003 and 2009. However, WaveMetrics played no role in the design, implementation or distribution of NeuroMatic, nor in the inception and writing of this manuscript. RAS declares that the research was conducted in the absence of any commercial or financial relationships that could be construed as a potential conflict of interest.
