# Supplementary material for: NeuroMatic: An Integrated Open-Source Software Toolkit for Acquisition, Analysis and Simulation of Electrophysiological Data
Source: Front Neuroinform. 2018 Apr 4;12:14. doi: 10.3389/fninf.2018.00014 (PMC5893720; doi:10.3389/fninf.2018.00014)
Supplement: Supplementary file 1 [file DataSheet1.pdf]

Supplementary Material

NeuroMatic: an integrated open-source software toolkit for acquisition, analysis and simulation of electrophysiological data

Jason S. Rothman* and R. Angus Silver

*** Correspondence:** Jason S Rothman: j.rothman@ucl.ac.uk

1 Supplementary Figures 1-7

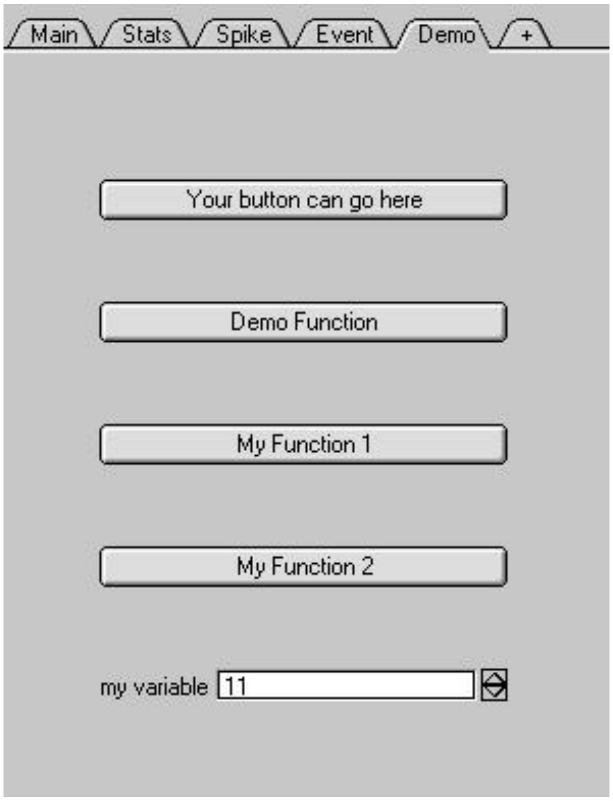

Supplementary Figure 1. NeuroMatic's Demo tab.

A simple NeuroMatic tab that provides a template for creating customized tabs and functions. Users can create their own tab by copying the Demo tab's procedure code (Menu/NeuroMatic/Procedures/Tabs/NM_DemoTab.ipf) to a new Igor procedure (Menu/Windows/New/Procedure...), replacing all instances of the word "Demo" with a short tab name (e.g. "FRAP") and saving the procedure file (Menu/File/Save Procedure As...). The new procedure can then be edited to suit the user's needs, including renaming or adding button and setvariable controls. The Demo tab's procedure code NM_DemoTab.ipf includes several demo functions (e.g. NMDemoLoop, NMDemoLoopThruChanWaves, NMDemoLoopThruFolders) which can be used as templates to create user-defined functions.

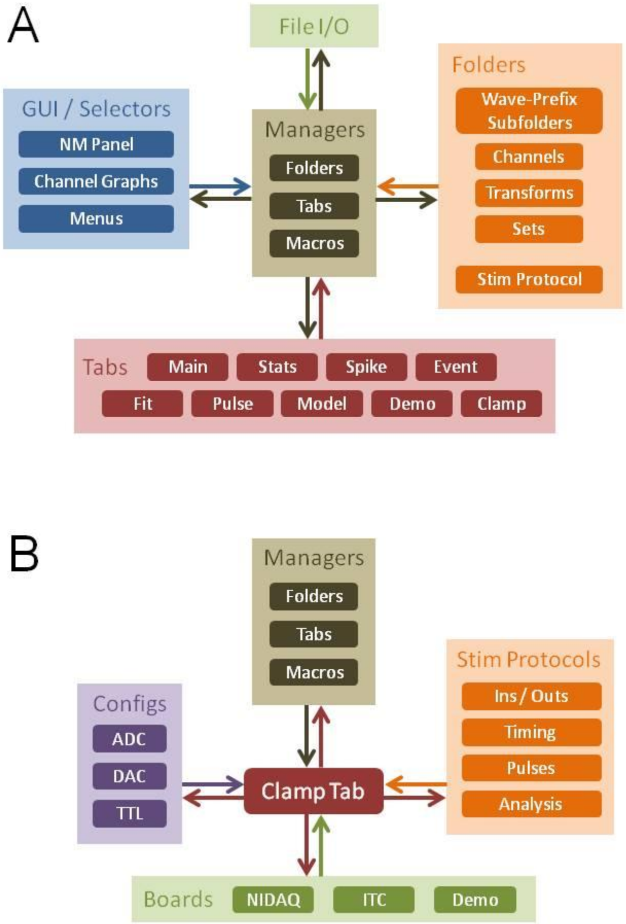

Supplementary Figure 2. NeuroMatic's software architecture.

(A) NeuroMatic has software managers for tabs, data folders, sets, channel graphs, analysis graphs/tables, history notes, configurations, data importing, fit functions, pulse waveforms, Hodgkin-Huxley-like models and "all waves" macro executions (**Supplementary Figure 3**). The managers coordinate the interaction between the NeuroMatic GUI controls, data folders, data importing/exporting and tool tabs. **(B)** Each tab is a manager in itself, where the Clamp tab is the most complicated, coordinating the interaction between user-defined stimulus protocols, ADC/DAC/TTL configurations, DAQ devices and online analysis.

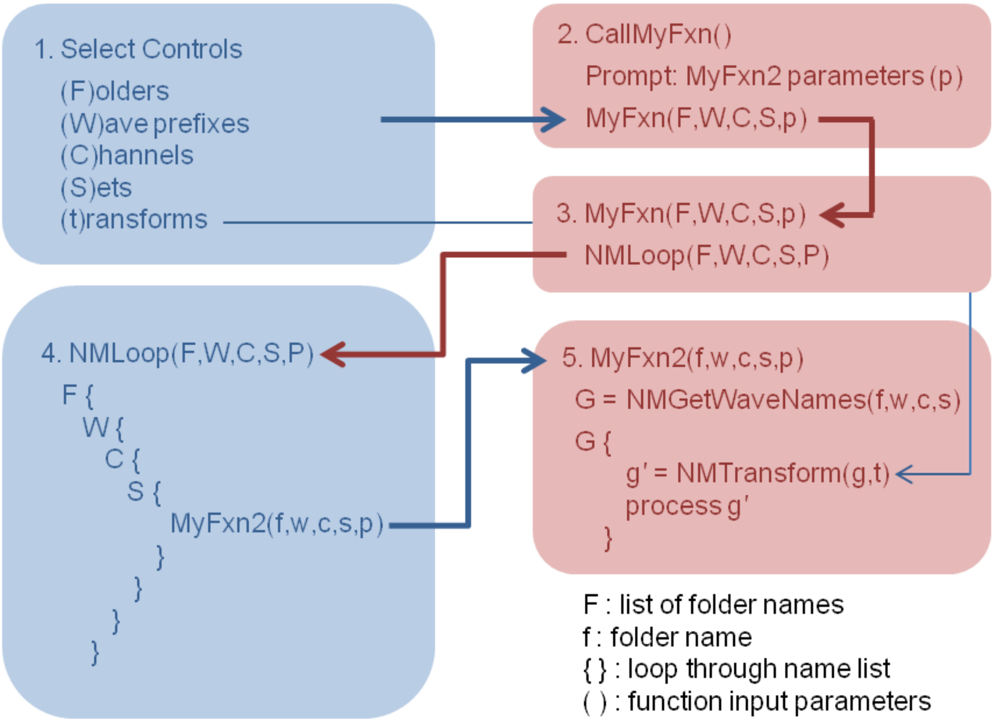

Supplementary Figure 3. Flowchart of NeuroMatic's "All Waves" function execution.

(1) Users select the data they wish to process via the *folder*, *wave-prefix*, *channel* and *wave/set selects* (**Figure 2**), and data transforms in the channel graphs (**Figure 3**). **(2)** Selecting a function on one of NeuroMatic's tabs executes a "call" function (e.g. CallMyFxn) that usually prompts the user for values of the function's input parameters (p_1, p_2, p_3...). The call function passes the select and input parameter values to a wrapper function **(3)**, e.g. MyFxn. The wrapper function does little except process and check the input parameters and pass them to NMLoop **(4)**, the workhorse of many NeuroMatic functions (Menu/NeuroMatic/Procedures/Misc/NM_Main.ipf/NMLoopExecute). NMLoop contains nested for-loops with a hierarchical structure of folders, wave prefixes, channels and sets. Each for-loop iterates over the items of its input list, passed as a semicolon-delimited string variable denoted with a capital letter (F, W, C, S). For example, the input sets list (S) might contain "Set1;Set2;". Inside the nested for-loops, individual parameter items (f, w, c, s, p) are passed to a wave processing function **(5)**, e.g. MyFxn2. Using the input parameters, the wave processing function obtains a list of waves to process (G) and, via a for-loop, performs the desired transformation and processing of these waves. Items in blue denote code and select controls at the core of NeuroMatic, whereas items in red denote top-level utility functions that have similar structure but perform different processing of the waves. Example code can be found in the Igor procedure file of NeuroMatic's Demo tab (Menu/NeuroMatic/Procedures/Tabs/NM_DemoTab.ipf/NMDemoLoop).

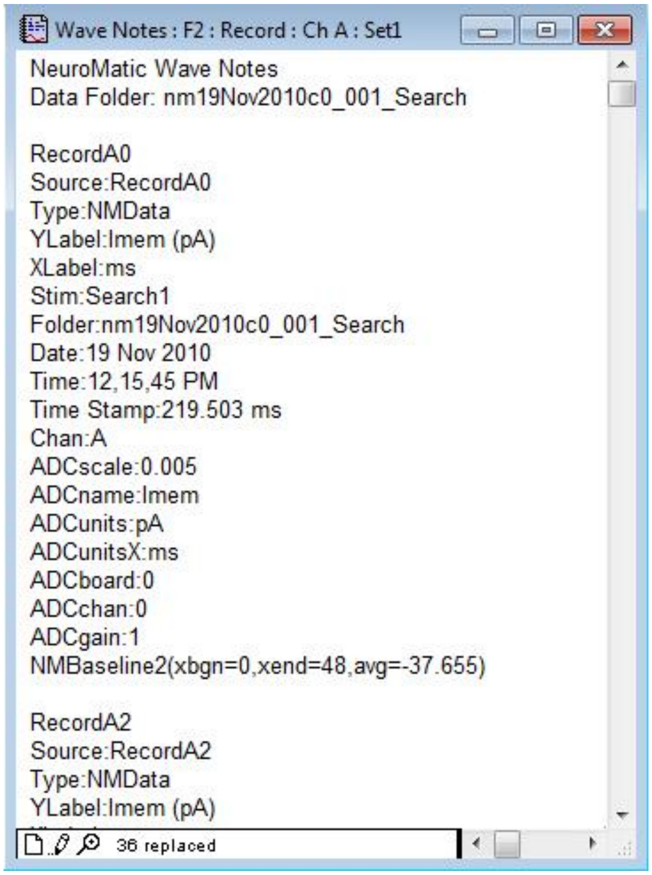

Supplementary Figure 4. NeuroMatic wave notes.

Screen capture of an Igor notebook displaying NeuroMatic waves notes (GUI/Main/Display/Print Notes) for wave “RecordA0” that include notes created by the Clamp tab during acquisition and the Main tab after applying a baseline transformation. The notebook lists the wave notes for 18 waves that are in Set1.

Global ADC Input Configurations

R0 | Imem

Config	ADCname	ADCunits	ADCboard	ADCchan	ADCscale	ADCmode
0	Imem	pA	0	0	0.01	
1	Vmem	mV	0	0	0.01	
2	Icmd	pA	0	1	0.001	
3	I_AMPA	pA	0	1	0.001	
4	I_GABA	pA	0	2	0.001	
5	I_NMDA	pA	0	3	0.001	
6	Temp	V	0	5	1	
7	Tmode	V	0	6	1	
8	Tgain_Axo	V	0	7	1	Tgain=B0_C0_Axopatch

Global DAC Input Configurations

R13 | 1

Config	DACname	DACunits	DACboard	DACchan	DACscale
0	Vcmd	mV	0	0	20
1	Icmd	pA	0	0	2000
2	G_AMPA	nS	0	1	2
3	G_GABA	nS	0	2	2
4	G_NMDA	nS	0	3	2
5	Stim	V	0	3	1

Supplementary Figure 5. Clamp tab configurations.

NeuroMatic's Clamp tab configurations for ADC inputs (top) and DAC outputs (bottom) viewed in table format (GUI/Clamp/DAQ/Table button) for an ITC DAQ device and Axopatch 200B patch-clamp amplifier. The configurations consist of a unique identification name (e.g. "Imem"), units (e.g. "pA"), scale factor as defined by the input/output channel of the amplifier (e.g. 0.01 V/pA) and the DAQ board number and channel number. When using one DAQ board, the board number should be 0. When using two DAQ boards (NIDAQ users only) the board numbers should be 0 or 1. The last ADC input configuration ("Tgain_Axo") is used for automatic scaling of the ADC input channel 0, using the telegraph gain output of the Axopatch 200B amplifier, which is connected to ADC input channel 7. The configuration was created by NeuroMatic after activating the "PreSamp/TeleGrph" checkbox on the Clamp/DAQ tab and selecting "Telegraph Gain", "Axopatch200B" and entering "0" for the ADC input channel to scale. Final Clamp tab configurations should be saved to disk via the "Save" button and opened for future use via the NeuroMatic menu (Menu/NeuroMatic/Configurations/Open).

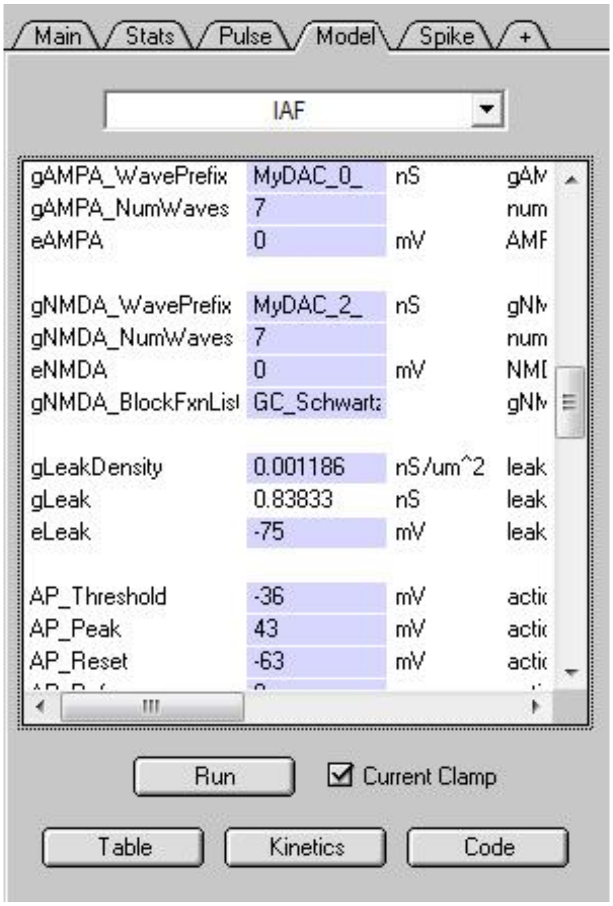

Supplementary Figure 6. NeuroMatic's Model tab.

Screen capture of NeuroMatic's Model tab configured to run an integrate-and-fire (IAF) model, with key parameters listed in a scrollable table for viewing and editing. Parameters were adjusted to replicate the GC in **Figure 14C**. The G_{AMPAR} and G_{NMDAR} waveforms used in the simulations (wave prefix names "MyDAC_0_" and "MyDAC_2_") were the same as those used to generate the dynamic-clamp recordings from this GC, created via the Pulse tab. The voltage-dependent activation equation of G_{NMDAR} was from (Schwartz et al., 2012; their Eq. 2). There was a tonic G_{GABAR} of 0.27 nS. After clicking the Run button, NeuroMatic generated 7 output waves with prefix name "Sim_Vmem" (see **Figure 15**). The waves were automatically selected for visualization and analysis.

Schwartz, E.J., Rothman, J.S., Dugué, G.P., Diana, M., Rousseau, C., Silver, R.A., et al. (2012). NMDA receptors with incomplete Mg^{2+} block enable low-frequency transmission through the cerebellar cortex. *J. Neurosci.* 32(20)**,** 6878-6893. doi: 10.1523/JNEUROSCI.5736-11.2012.

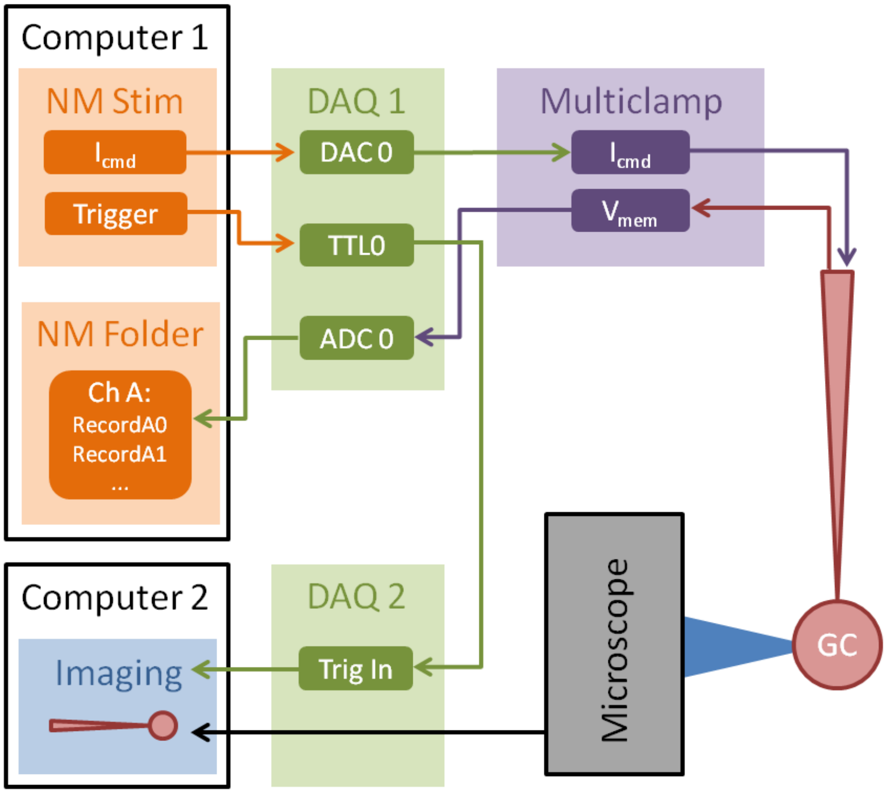

Supplementary Figure 7. NeuroMatic patch-clamp acquisition with simultaneous imaging.

On computer #1, a NeuroMatic stimulus protocol (master) is configured to perform standard patch-clamp acquisition via DAQ device #1 and a Multiclamp amplifier. The protocol also sends a TTL pulse to the triggered input line of DAC device #2, which triggers imaging acquisition on computer #2 (slave). Note that NeuroMatic can also act as the slave during triggered acquisition (GUI/Clamp/Stim/Time/epic triggered or continuous triggered).
